# A plant-specific cytochrome *b_5_*–like protein is essential for phytosterol biosynthesis

**DOI:** 10.1126/sciadv.ady1719

**Published:** 2025-09-10

**Authors:** Xianhai Zhao, Lijun Qiao, Zhi-Yong Wang, Shou-Ling Xu, John Shanklin, Chang-Jun Liu

**Affiliations:** ^1^ Biology Department, Brookhaven National Laboratory, Upton, NY 11973, USA.; ^2^ Carnegie Institution for Science, Stanford, CA 94305, USA.

## Abstract

Sterols are essential isoprenoid derivatives that contribute to membrane structure and function. In plants, they also serve as precursors to phytohormones and specialized metabolites important for development, defense, and health. Although the sterol biosynthetic pathway is considered well-characterized, we report the discovery of a plant-specific cytochrome *b_5_*–like protein, CB5LP, as a critical component of phytosterol biosynthesis. Loss of CB5LP in *Arabidopsis* causes embryonic defects, seedling lethality, and accumulation of 14α-methyl-sterols, with reduced levels of sitosterol and stigmasterol—indicating a defect in sterol 14α-demethylation. TurboID-based proximity labeling and in vitro assays show that CB5LP physically and functionally interacts with CYP51, a cytochrome P450 enzyme catalyzing this demethylation step. Unlike canonical cytochrome *b_5_* proteins, CB5LP has a reversed topology and is exclusive to plants, acting as an evolutionarily distinct electron donor. This discovery reveals an uncharacterized redox partnership essential for sterol biosynthesis and highlights a promising target for the development of selective herbicide.

## Teaser

A plant-specific CB5-like protein as CYP51 redox partner is essential for sterol biosynthesis and a potential herbicide target.

## INTRODUCTION

Sterols are isoprenoid-derived molecules essential to eukaryotic plasma membrane. Different organisms synthesize distinct sterols via the conserved squalene pathway, with fungi producing ergosterols, animals and insects producing cholesterol, while higher plants synthesizing diverse phytosterols such as sitosterol, stigmasterol, and campesterol (*1*–*3*). Despite their varied origins, these sterols share chemical similarities, differing primarily by a few carbon atoms—cholesterol (C-27), ergosterols, and phytosterols (C-28 and C-29)—highlighting evolutionary divergence in sterol biosynthesis (*4*).

Sterols play a critical role in membrane dynamics, maintaining fluidity and supporting essential biological processes (*5*). Phytosterols, in particular, exhibit diverse biological functions in plants; for example, campesterol serves as a precursor for brassinosteroids, a class of phytohormones influencing growth and stress responses (*6*); sitosterol-β-glucoside aids cellulose synthesis (*7*); and sterol composition affects vascular cell polarization (*8*). Cholesterol in plants also contributes to the synthesis of specialized metabolites like α-solanine and α-tomatine that enhance plant defense mechanisms (*9*). Beyond their biological roles in plants, phytosterols offer notable dietary health benefits. They exhibit antioxidant, anticancer, antidiabetic, and cholesterol-lowering properties, with 7-dehydrocholesterol (provitamin D3) being a key precursor for vitamin D3 (*10*, *11*). Given their beneficial health properties, phytosterols are receiving increased attention as dietary supplements for the prevention of human diseases (*11*–*13*).

Phytosterol biosynthesis follows the post–squalene pathway, in which cycloartenol as a primary biosynthetic intermediate undergoes multiple modifications, such as C-24 methylation, C-4 decarboxylation, C-14 demethylation, C-5 desaturation, etc., leading to the formation of key phytosterols sitosterol, stigmasterol, and campesterol (fig. S1) (*3*, *9*). These modifications, catalyzed by specific enzymes, shape the diverse phytosterol profile in plants. Among the cytochrome P450 enzymes involved, cytochrome P450 subfamily 51 (CYP51) is the most upstream and functionally critical enzyme in phytosterol biosynthesis. CYP51s catalyze the essential 14α-demethylation step in biosynthesis of sterols by three consecutive hydroxylations at the C14 position followed by elimination of formic acid. The introduction of the double bound is essential as it serves to flatten the sterol backbone structure to enable proper insertion into membranes. CYP51 is the only P450 family conserved across evolution within fungi, animals, and plants, in which it plays a universal role in sterol biosynthesis (*14*, *15*) and is essential for the survival of yeasts, fungal pathogens, mice, and plants (*16*–*19*). CYP51 has therefore emerged as the main target for developing fungicides and pesticides for therapeutic and agricultural applications (*20*, *21*).

In plant, CYP51 encodes obtusifoliol 14α-demethylase and is functionally characterized in maize, sorghum, wheat, *Arabidopsis*, and rice (*18*, *22*–*26*). The *Arabidopsis* genome contains two *CYP51* genes, *CYP51A1* and *CYP51A2*, with the former being an expressed pseudogene (*18*). The null mutant of *Arabidopsis CYP51A2* gene is seedling-lethal and accumulates obtusifoliol, the substrate of CYP51 (*18*). In contrast, fungal CYP51 enzymes use lanosterol as a substrate. This biochemical discrepancy has led to the development of azole drugs specifically targeting fungal CYP51 for use in both agriculture and medicine (*18*, *20*, *21*). However, the emergence of azole-resistant fungal strains poses a substantial challenge to public health and agriculture (*21*). A deeper understanding of CYP51 catalytic and regulatory mechanisms is crucial for developing novel CYP51 inhibitors.

Endoplasmic reticulum (ER) membrane–resident cytochrome P450s recruit membrane-bound redox partner(s) to deliver electrons from pyridine dinucleotide cofactors NADPH (reduced form of nicotinamide adenine dinucleotide phosphate) and/or NADH [reduced form of nicotinamide adenine dinucleotide (oxidized form)] to support their catalysis. The eukaryotic ER membrane system contains two electron transfer chains; one involves NADPH-dependent cytochrome P450 oxidoreductase (CPR), and another is composed of NADH-dependent cytochrome *b_5_* reductase (CBR) and cytochrome *b_5_* (CB5) (fig. S2). CPR is considered the primary electron donor for various P450 enzymes (*27*), although, in some cases, CB5 can functionally associate with its cognate reductase CBR or CPR, to shuttle electrons to P450s (*28*). *Arabidopsis* genome has five annotated *CB5* genes, *AtCB5A* (At1g26340), *AtCB5B* (At2g32720), *AtCB5C* (At2g46650), *AtCB5D* (At5g48810), and *AtCB5E* (At5g53560), all of which encode the tail-anchored proteins with an N-terminal heme–binding (cyt-*b_5_*) domain and a C-terminal transmembrane (TM) domain (*29*). Among them, AtCB5D has been identified to play a critical electron donor role in syringyl lignin biosynthesis (*30*). In addition to these CB5s, *Arabidopsis* has a CB5-like protein (AtCB5LP, At1g60660), which shares 36 to 46% amino acid sequence identity with canonical CB5s (fig. S3, A and B), but exhibits reversed topology, with its TM domain at the N terminus and cyt-*b_5_* domain at the C terminus (fig. S3C). This unique structural arrangement distinguishes AtCB5LP from others within the CB5 family; however, its biochemical and biological functions remain unknown.

In the present study, we establish that CB5LP is critical for 24-ethylidene phytosterol biosynthesis. Its depletion in *Arabidopsis* causes embryonic defects and postembryonic lethality, resembling the phenotypes caused by disruption of *CYP51A2*. The loss of CB5LP results in the increased accumulation of 14α-methyl-sterols and reduced levels of sitosterol and stigmasterol, highlighting its pivotal role in sterol 14α-demethylation. In vitro assay using extracted yeast microsomes reveals that CB5LP is essential for supporting CYP51A2 catalytic activity, while its mutant variant, devoid of electron transfer ability, shows no effect on CYP51 function. Notably, unlike canonical CB5 proteins, CB5LP homologs are exclusive to plant lineages and are absent in fungi and mammals. This finding establishes CB5LP as a plant-specific, structurally unique electron donor essential for CYP51-mediated phytosterol biosynthesis.

## RESULTS

### Disruption of *AtCB5LP* results in plant growth defects

The annotated *Arabidopsis CB5LP* gene has a coding sequence of 366–base pair (bp) nucleotides, encoding a 121–amino acid polypeptide with a predicted molecular weight of 13.7 kDa and an Isoelectric point (PI) of 5.46. Unlike canonical CB5 proteins, CB5LP has a reversed topology, bearing an N-terminal TM domain and a C-terminal cyt-*b_5_* domain (fig. S3C).

**Fig. 1.**
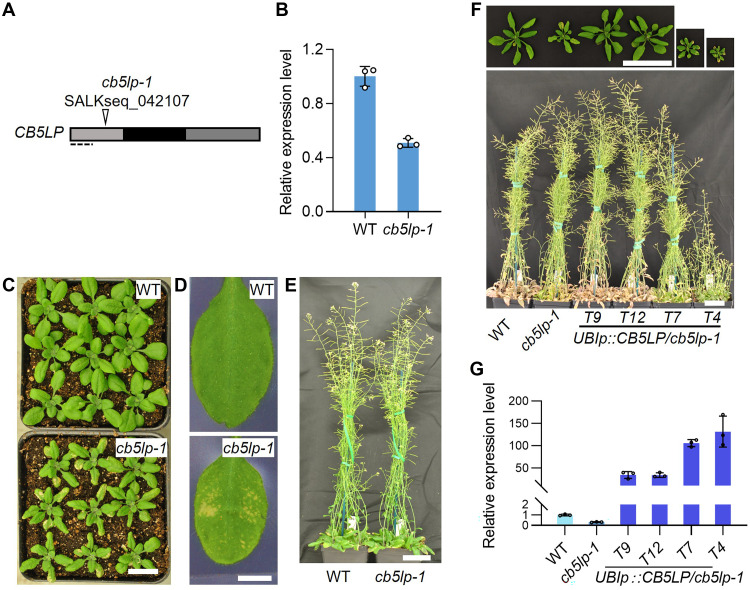
Characterization of the *cb5lp-1* mutant. (**A**) T-DNA insertion site in the *cb5lp-1* mutant, indicated with triangle. The dashed line delineates the region used for RT-qPCR analysis. The gray boxes indicate 5′ and 3′ untranslated regions, while black box indicates the single exon of *CB5LP*. (**B**) Relative expression levels of *CB5LP* in 1-week-old seedlings of the WT (Col-0) and *cb5lp-1* mutant, examined via RT-qPCR. Data are presented as means ± SD from three biological replicates. (**C**) Morphology of 3-week-old WT and *cb5lp-1* plants in soil. (**D**) Close-up views of the rosette leaves from 3-week-old plants of WT and *cb5lp-1* mutant, highlighting the yellowish areas on *cb5lp-1* leaves. (**E**) Morphology of 6-week-old WT and *cb5lp-1* plants. (**F**) Morphology of 4-week-old (top) and 2-month-old (bottom) WT, *cb5lp-1*, and four transgenic lines. (**G**) Relative expression levels of *CB5LP* in 1-week-old seedlings of WT, *cb5lp-1* mutant, and four independent transgenic lines, examined via RT-qPCR. CB5LP expression level in WT was set as 1. Data are presented as means ± SD from three biological replicates. Scale bars, 2 cm (C), 5 mm (D), 10 cm (E), and 5 cm (F).

To investigate its biological functions, we obtained a T-DNA insertion line of the *AtCB5LP* gene, designated *cb5lp-1* (SALKseq_042107), from the Arabidopsis Biological Resource Center (ABRC). Its T-DNA insertion is in the 5′ untranslated region (Fig. 1A). Reverse transcription quantitative polymerase chain reaction (RT-qPCR) analysis confirmed the isolated homozygous *cb5lp-1* as a knockdown mutant, which retains approximately 50% of *AtCB5LP* transcript levels to the WT (Fig. 1B).

The 3-week-old *cb5lp-1* plants grown in soil showed smaller stature, with yellowish spots spread on their rosette leaves, compared to the wild type (WT) (Fig. 1, C and D). As they matured, *cb5lp-1* plants gradually regained development and eventually resembled the WT (Fig. 1E). These data suggest that *AtCB5LP* down-regulation leads to developmental disturbance in *Arabidopsis*. To validate this, we introduced *AtCB5LP* transgene, driven by the *Arabidopsis UBIQUITIN-10* gene promoter (*UBIp*), into the *cb5lp-1* mutant. The obtained transgenic lines, such as T9 and T12, successfully restored the WT-like rosette leaf stature and morphology, confirming that *AtCB5LP* expression rescues *cb5lp-1* defective phenotypes (Fig. 1F). A few transgenic lines, such T7 and T4, exhibited more severe growth defects than *cb5lp-1*, with even smaller rosette leaves and severe dwarfism (Fig. 1F). Expression analysis revealed higher *AtCB5LP* transcript levels in T7 and T4 lines compared to the T9 and T12 lines (Fig. 1G). This suggests that the precise *AtCB5LP* expression level is critical for sustaining plant growth, as excessive accumulation of *CB5LP* transcripts is likely detrimental to *Arabidopsis* development.

**Fig. 2.**
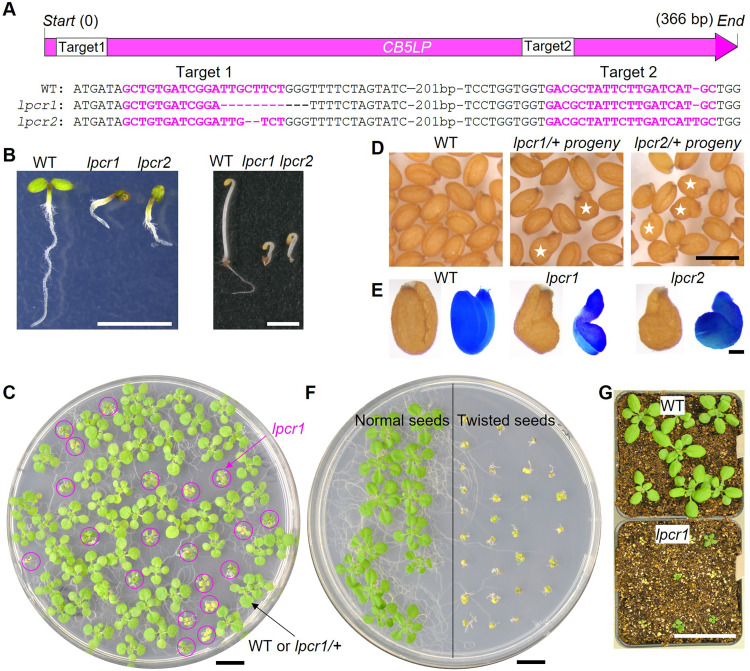
Characterization of *CB5LP* CRISPR-Cas9 knockout mutants. (**A**) Schematic representation of the *CB5LP* gene showing the two CRISPR target sites and their corresponding mutations in the knockout *lpcr* mutants, *lpcr1* and *lpcr2*. (**B**) Morphology of 4-day-old WT and *lpcr* mutant seedlings grown under 16-hour light/8-hour dark (left) and continuous dark (right) conditions. (**C**) Morphology of 2-week-old *lpcr1/+* progeny seedlings. Homozygous *lpcr1* mutant seedlings segregated from the *lpcr1/+* parent lines are circled. (**D**) Morphology of the dried mature seeds of *lpcr1/+* progeny. White stars indicate seeds with a twisted shape. (**E**) Morphology of seeds with (left) and without (right) seed coats. After seed coat removal, embryos were stained with toluidine blue for observation. (**F**) Three-week-old seedlings germinated from normal (left) and twisted (right) seeds of *lpcr1/+* progeny on 1/2 MS medium. (**G**) Morphology of 3-week-old WT and *lpcr1* plants in soil. Scale bars, 2 mm (B), 1 cm [(C) and (F)], 0.5 mm (D), 0.1 mm (E), and 5 cm (G).

To further validate CB5LP function, we used CRISPR-Cas9 gene editing to knock out *CB5LP* in *Arabidopsis* (fig. S4). Genotyping 36 T1 generation of transgenic plants via Sanger sequencing, however, revealed no homozygous knockout lines. Instead, we identified two heterozygous lines, *lpcr1/+* and *lpcr2/+*. The *lpcr1* line harbors an 11-bp deletion, while *lpcr2* carries a 2-bp deletion and a 1-bp insertion in the *CB5LP* gene (Fig. 2A and fig. S4A).

Culturing *lpcr/+* progeny on half-strength Murashige and Skoog agar medium (1/2 MS) under both light and dark conditions resulted in the emergence of a number of smaller seedlings with shorter roots and hypocotyl (Fig. 2, B and C), which were confirmed as the segregated *lpcr* homozygous lines (fig. S4B). Examining *lpcr/+* progeny seeds under a dissection microscope, we observed a number of smaller, shriveled, and twisted seeds (Fig. 2D). Upon seed coat removal, we found that the enclosed embryos displayed L- or Z-shaped, distinct from the normal U-shaped WT embryos, indicating embryonic defects in the *lpcr* mutant line (Fig. 2E). These deformed seeds germinated into tiny seedlings on the 1/2 MS medium (Fig. 2F), which were verified as homozygous *CB5LP* mutants via Sanger sequencing (fig. S4C). However, these smaller seedlings failed to grow further and died within a month, both on 1/2 MS medium and in soil (Fig. 2, F and G), suggesting that *CB5LP* knockout is seedling lethal. All these data underscore the critical role of CB5LP in *Arabidopsis* seed and seedling development. To remove the T-DNA insertion, we backcrossed *lpcr1/+* with Col-0. After PCR genotyping to identify Cas9-free lines and Sanger sequencing to confirm the presence of the *lpcr1* allele in F2 individuals, we further validated the absence of T-DNA in the result mutant lines by hygromycin antibiotic–sensitivity assay on F3 generation plants (fig. S4, D to F). Through this process, we successfully obtained a T-DNA–free *lpcr1/+* mutant, which was used for subsequent studies.

### CB5LP is an ER-localized protein

**Fig. 3.**
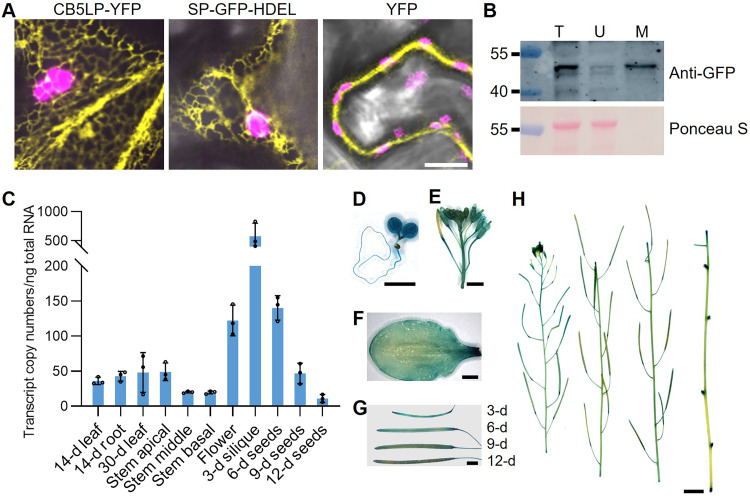
Subcellular localization and expression pattern of CB5LP. (**A**) Representative fluorescence pattern of CB5LP-YFP, SP-GFP-HDEL ER marker, and free YFP in *N. benthaniana* leaf epidermal cells. Yellow pseudo-color indicates YFP or GFP fluorescence signals, and magenta indicates chloroplast autofluorescence. (**B**) Immunoblots of CB5LP-YFP in the total (T), upper phase (U), and microsomal phase (M) fractions. Rubisco large subunit stained by Ponceau S was used as cytosol phase indicator. The experiments in (A) and (B) were repeated three times independently, yielding similar results. (**C**) RT-qPCR analysis of absolute *CB5LP* transcript levels in the indicated *Arabidopsis* tissues. Young leaves and roots were collected from 14-day-old seedlings, mature leaves from 30-day-old plants, and stems and flowers from 50-day-old plants. Siliques and seeds were collected at the indicated day after anthesis. Tissues from at least five individual plants were pooled as one biological replicate. Data are presented as means ± SD from three biological replicates. (**D** to **H**) GUS staining analysis of *CB5LPp::GUS* transgenic plants in (D) 7-day-old seedling, (E) flower, (F) rosette leaf, (G) siliques, and (H) 50-day-old inflorescence. Scale bars, 10 μm (A), 2 mm [(D) to (G)], and 1 cm (H). d, day.

Canonical CB5 proteins exhibit different subcellular localizations. While AtCB5A targets chloroplasts or mitochondria, the other four (AtCB5B to AtCB5E) are the ER localized (*29*, *31*). To examine subcellular localization of CB5LP, we transiently expressed AtCB5LP fused with yellow fluorescent protein (YFP) in *Nicotiana benthamiana* leaf epidermal cells. The distribution of fluorescent signals displayed a characteristic ER network pattern, resembling that of the SP–green fluorescent protein (GFP)–HDEL ER marker protein (*32*, *33*) (Fig. 3A). Furthermore, we prepared microsomal membrane fraction from *N. benthamiana* leaves expressing CB5LP-YFP. Immunoblotting detected CB5LP exclusively in the endomembrane fraction, while the Rubisco large subunit remained in the cytosolic phase (Fig. 3B). These results demonstrate that AtCB5LP, like canonical AtCB5B-E, is an ER-localized protein.

### *CB5LP* is ubiquitously expressed in plant tissues

Subsequently, *CB5LP* expression patterns were examined via RT-qPCR. As depicted in Fig. 3C, *CB5LP* transcripts were detected in all examined tissues, including seedling leaves and roots, rosette leaves, stems, flowers, siliques, and seeds. Among these, relatively high transcript levels were observed in developing siliques, young seeds, and flowers (Fig. 3C). In addition, we generated transgenic lines expressing *GUS* reporter gene under the control of the *CB5LP* promoter (*LPp*) (data S1). GUS staining assays revealed strong signals in all examined tissues, including cotyledon leaves, seedling roots, rosette leaves, stems, flowers, and siliques (Fig. 3, D to H). This ubiquitous gene expression pattern suggests that CB5LP serves a potential housekeeping function, playing a role in different plant tissues and developmental stages.

### CB5LP has electron transfer ability crucial for its function and plant growth

**Fig. 4.**
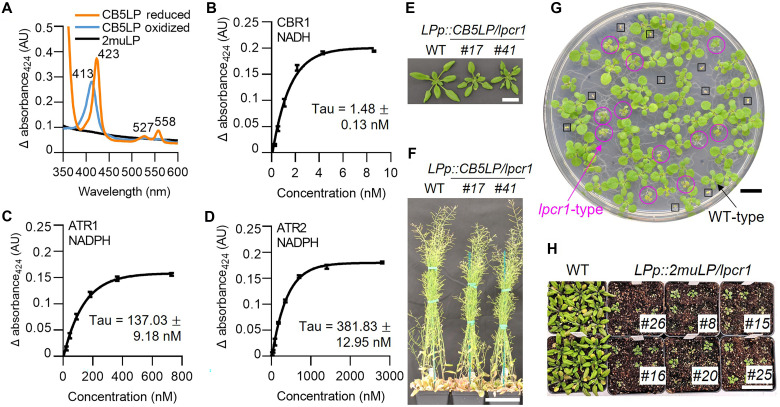
Evaluation of CB5LP redox properties. (**A**) Absolute absorption spectra of recombinant CB5LP (9 μM) in its oxidized (blue line) and sodium dithionite–reduced (orange line) forms. The recombinant mutant variant 2muLP (9 μM) was used as a control and showed no Soret band absorption. (**B** to **D**) Monophasic reduction kinetics of CB5LP by CBR1 (B), ATR1 (C), or ATR2 (D) at 60 s, plotted as a function of increasing reductase concentrations. Recombinant CB5LP (9 μM) was incubated with the indicated reductase at room temperature. Reactions were initiated by the addition of 100 μM NADH (B) or 100 μM NADPH (C and D). (**E** and **F**) Morphology of 4-week-old (E) and 8-week-old (F) *LPp::CB5LP/lpcr1* T2 transgenic plants. (**G**) Morphology of 2-week-old T2 progeny of the *LPp:2muLP/lpcr1/+ #16* transgenic line grown on hygromycin-containing selective medium. The WT-type seedlings harboring *LPp::2muLP* transgene in the segregated WT or *lpcr1/+* background appear normal. The *lpcr1*-type seedlings carrying *LPp::2muLP* transgene in the segregated *lpcr1* background exhibit hygromycin resistance but smaller stature, as indicated with circles. Hygromycin-sensitive nontransgenic seedlings are boxed. (**H**) Growth phenotype of 4-week-old *lpcr1-type LPp::2muLP/lpcr1* T2 plants in soil. Scale bars, 2 cm (E), 10 cm (F), 1 cm (G), and 5 cm (H).

CB5LP contains a heme-binding domain at its C terminus. To determine whether CB5LP retains electron transfer properties like canonical CB5s, we expressed a truncated recombinant CB5LP protein (lacking N-terminal TM) in *Escherichia coli* and assessed its redox properties. In addition, we generated a mutant variant, 2muLP, in which two histidine residues, His^81^ and His^104^, predicted for heme binding based on AlphaFold homology modeling, were substituted with alanine, thus potentially disrupting its heme-binding and electron transport ability (fig. S5, A and B). This 2muLP was included as the control. Upon isopropyl-β-d-thiogalactopyranoside (IPTG) induction, the collected CB5LP-expressing *E. coli* pellets exhibited a reddish tint, indicating accumulation of holo-CB5 protein with heme binding (fig. S5C). In contrast, the 2muLP-expressing cells appeared pale yellow, indicative of heme-binding loss (fig. S5C). Subsequently, the purified recombinant proteins confirmed these observations: CB5LP retained a reddish tint, whereas 2muLP appeared transparent (fig. S5, D and E). Spectroscopic analysis of purified CB5LP showed a characteristic Soret peak at 412 nm, signifying its heme-bound state (fig. S5F). Addition of sodium dithionite caused an absorption shift from 413 nm (oxidized form) to 423 nm (characteristic of the reduced form), along with the appearance of additional absorption peaks at 527 and 558 nm (Fig. 4A), consistent with the expected redox electrochemical properties of the recombinant CB5LP. In contrast, the recombinant 2muLP did not exhibit any characteristic absorption under the same treatments (Fig. 4A).

To further assess CB5LP’s redox capability, we incubated it with *Arabidopsis* reductase CBR1, ATR1, and ATR2. Like the canonical CB5 proteins (*34*), CB5LP was effectively reduced by CBR1 in the presence of NADH and by ATR1 or ATR2 in the presence of NADPH at their native concentrations (Fig. 4, B to D). The reductase incubations completely converted oxidized CB5LP to its reduced form within 5 min, as evidenced by the diagnostic shift of its Soret band from 413 to 424 nm (Fig. 4, B to D). Consistent with the findings from the canonical AtCB5D (*34*), a higher concentration of ATR1 or ATR2 was required than CBR1 to fully reduce the same amount of oxidized CB5LP. The calculated Tau values were 137 nM for ATR1 and 381 nM for ATR2, compared to 1.48 nM for CBR1, indicating that CBR1 is more kinetically efficient at reducing CB5LP (Fig. 4, B to D).

To assess the functional significance of CB5LP’s electron transfer ability, we introduced the WT CB5LP and its mutant variant 2muLP, respectively, into the *lpcr1* mutant. To avoid the observed developmental defects caused by the hyperaccumulation of *CB5LP* transcripts driven by *UBIp* (Fig. 1, F and G), the native *LPp* promoter was used (data S1). Because of the seedling lethality of homozygous *lpcr1* mutant, the CRISPR-Cas9 T-DNA–free heterozygous *lpcr1/+* line was used as the parent for gene transformation. PCR genotyping and Sanger sequencing were used to identify transformants in a homozygous *lpcr1* or heterozygous *lpcr1/+* background in the T1 generation. For *LPp::CB5LP* transgenic events, if *CB5LP* functions properly, it should rescue the development defects of the homozygous *lpcr1*, allowing transgenic seedlings to reach maturity and produce seeds. After confirming the homozygosity of the *lpcr1* background and the proper expression of *CB5LP* transgene in the *LPp::CB5LP* transgenic plants (fig. S6, A and B), we observed that the *LPp::CB5LP* transgenic plants indeed displayed normal growth, indistinguishable from the WT, indicating successful complementation of the *lpcr1* mutants by *CB5LP* (Fig. 4, E and F).

For the *LPp::2muLP* complementation assay, considering the possibility that *LPp::2muLP* might fail to complement the *lpcr1* mutation, we first identified T1 transgenic lines carrying the *lpcr1* allele through PCR genotyping (fig. S6C) and selected six representative transgenic lines in a heterozygous *lpcr1/+* background, as confirmed by Sanger sequencing (fig. S6D). These plants were able to grow to maturity and produce T2 seeds. The T2 segregants of the *LPp::2muLP* transgenic lines, when grown on antibiotic-containing selective media, displayed two distinct seedling phenotypes: normal sized, WT-type seedlings, representing segregants harboring the *LPp::2muLP* transgene in either a WT or heterozygous *lpcr1/+* background; and smaller, *lpcr1-type* seedlings, indicative of segregants in a homozygous *lpcr1* background that inherited the *LPp::2muLP* transgene from *lpcr1/+* parents (Fig. 4G). Sanger sequencing confirmed the *lpcr1* homozygous genotype, while RT-PCR and RT-qPCR analyses verified the optimal expression of the *2muLP* transgene in these seedlings (fig. S6, E to G). Notably, *lpcr1-type* T2 segregants were consistently observed in all 40 independent *LPp::2muLP* transgenic lines screened. When transferred to soil, these small *lpcr1*-type seedlings failed to develop into mature plants, recapitulating the authentic *lpcr1* mutant phenotype (Fig. 4H). This finding indicates that the 2muLP variant indeed fails to complement the CB5LP deficiency. These results collectively demonstrate that sustaining electron transfer via CB5LP is essential for plant growth, as the electron transfer–deficient 2muLP variant could not substitute for CB5LP in *lpcr1* mutants.

### CB5LP potentially associates with diverse P450s

**Fig. 5.**
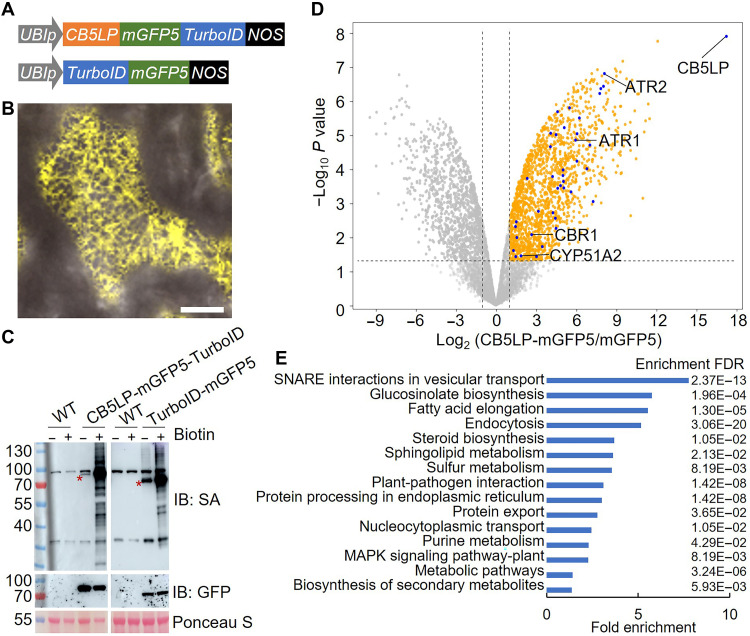
Identification of proteins proximal to CB5LP. (**A**) Schematic representation of plant expression cassettes used for proximity labeling experiments. (**B**) Fluorescence distribution pattern of CB5LP-mGFP5-TurboID in *Arabidopsis* cotyledon cells. Yellow pseudo-color indicates fluorescence signals. Scale bar, 10 μm. (**C**) Immunoblots showing biotinylated proteins from *CB5LP-mGFP5-TurboID* and *TurboID-mGFP5* transgenic plants, treated (+) or untreated (−) with exogenous biotin, detected with streptavidin (SA) and anti-GFP antibodies. Asterisks mark the positions of CB5LP-mGFP5-TurboID and TurboID-mGFP5 fusion proteins. (**D**) Volcano plot depicting significantly enriched proteins (log_2_ fold change ≥1 and –log_10_ *P* ≥ 1.3) between CB5LP-mGFP5-TurboID and TurboID-mGFP5 samples. Significantly enriched proteins are highlighted in orange; CB5LP, ATR1, ATR2, CBR1, and the 36 enriched P450s are highlighted in blue. (**E**) GO pathway enrichment analysis of 1676 proteins enriched in the CB5LP-TurboID assay, showing the top 15 biological pathways.

Given the embryonic and postembryonic defects observed in *lpcr* mutant, we sought to uncover the molecular mechanisms underlying plant lethality. Since CB5LP functions as an electron transfer protein (Fig. 4), we hypothesized that it may physically associate with and transfer electrons to specific redox partners involved in key biological processes. To explore CB5LP-associated proteins, we used TurboID-based protein proximity labeling coupled with mass spectrometry analysis. TurboID, an engineered biotin ligase, hydrolyzes adenosine 5′-triphosphate to activate biotin and produce reactive biotinyl–adenosine 5′-monophosphate that covalently labels proximal protein lysine residues within 10 nm. Biotinylated proteins are then isolated using streptavidin-coated beads and identified via mass spectrometry (*35*). We generated stable transgenic lines of *Arabidopsis* expressing *CB5LP-mGFP5-TurboID* and *TurboID-mGFP5* (as a control), respectively (Fig. 5A). Fluorescence imaging confirmed the ER localization of CB5LP-mGFP5-TurboID fusion protein, indicating its proper physiological function in transgenic plants (Fig. 5B). After a 3-hour treatment of transgenic seedlings with 100 μM biotin, abundant biotin-labeled protein bands were visualized from TurboID-expressing plants, validating the efficacy of the CB5LP-TurboID system (Fig. 5C).

Using label-free quantification via liquid chromatography–tandem mass spectrometry (LC-MS/MS), we identified 1676 enriched CB5LP-binding candidates (data S2). Among them, three reductases, CBR1, ATR1, and ATR2, known to reduce CB5LP, were detected, validating the TurboID approach. Gene ontology (GO) pathway enrichment revealed that the proteins of the CB5LP proxiome are potentially involved in a variety of biological processes including soluble *N*-ethylmaleimide–sensitive factor attachment protein receptor–mediated vesicular trafficking, glucosinolate biosynthesis, sterol biosynthesis, and sphingolipid biosynthesis, etc. (Fig. 5E and data S3). Notably, among the 1676 enriched proteins, 36 are cytochrome P450 enzymes (Fig. 5D and data S2). GO pathway analysis suggested that these P450s are potentially involved in the biosynthesis of glucosinolates, steroids, sphingolipids, purines, cutin, suberin, waxes, and phenylpropanoids (data S4). Subsequently, we conducted broad metabolic profiling on *CB5LP* mutants to investigate the metabolic impacts of CB5LP.

### CB5LP has marginal effects on phenolics, lipids, and glucosinolates biosynthesis

Metabolic analysis of phenylpropanoids of *lpcr1* seedlings revealed only a slight decrease in leaf sinapoyl esters level but substantially increased accumulation of kaempferol and anthocyanins (fig. S7, A to C). The enhancement of flavonoid and anthocyanins accumulation levels likely results from a secondary stress response due to growth defects, which is reminiscent of lignin-deficient dwarf plants such as *hct* or *ref8* (*36*). Lipid profiling of *lpcr1* seedlings and seeds, assessed via thin-layer chromatography (TLC) and/or gas chromatography–mass spectrometry (GC-MS), showed no obvious changes in their wax content and most fatty acid species, compared to WT, except for hexadecatrienoic acid (C16:3) in *lpcr1* seedlings and α-linolenic acid (C18:3) in *lpcr1* seeds, which exhibited a slight reduction (fig. S7, D to F).

Given that CB5LP proximity labeling identified a few glucosinolate biosynthetic P450 enzymes (data S3), we examined glucosinolate composition in 14-day-old seedlings of *lpcr1*. The results showed that the disruption of *CB5LP* significantly reduced the levels of butyl glucosinolates, including 4-methylsulfinylbutyl (4MSOB) and 4-methylthiobutyl (4MTB) glucosinolates while enhancing octyl- and indo-glucosinolate production (figs. S7 and S8). This suggests a potential functional association of CB5LP with aliphatic glucosinolate biosynthesis. Nevertheless, deficiency in herbivory- or pathogen-defense glucosinolate biosynthesis is not typically associated with embryo lethality of *Arabidopsis* (*37*), which contrasts with the seedling lethality observed in *lpcr1*.

Collectively, these data suggest that *CB5LP* mutants displayed some direct or secondary effects on the synthesis of phenylpropanoids, fatty acids, and glucosinolates. However, none of these metabolic changes are sufficient to explain the seedling lethality of the mutants.

### CB5LP is essential for phytosterol biosynthesis

**Fig. 6.**
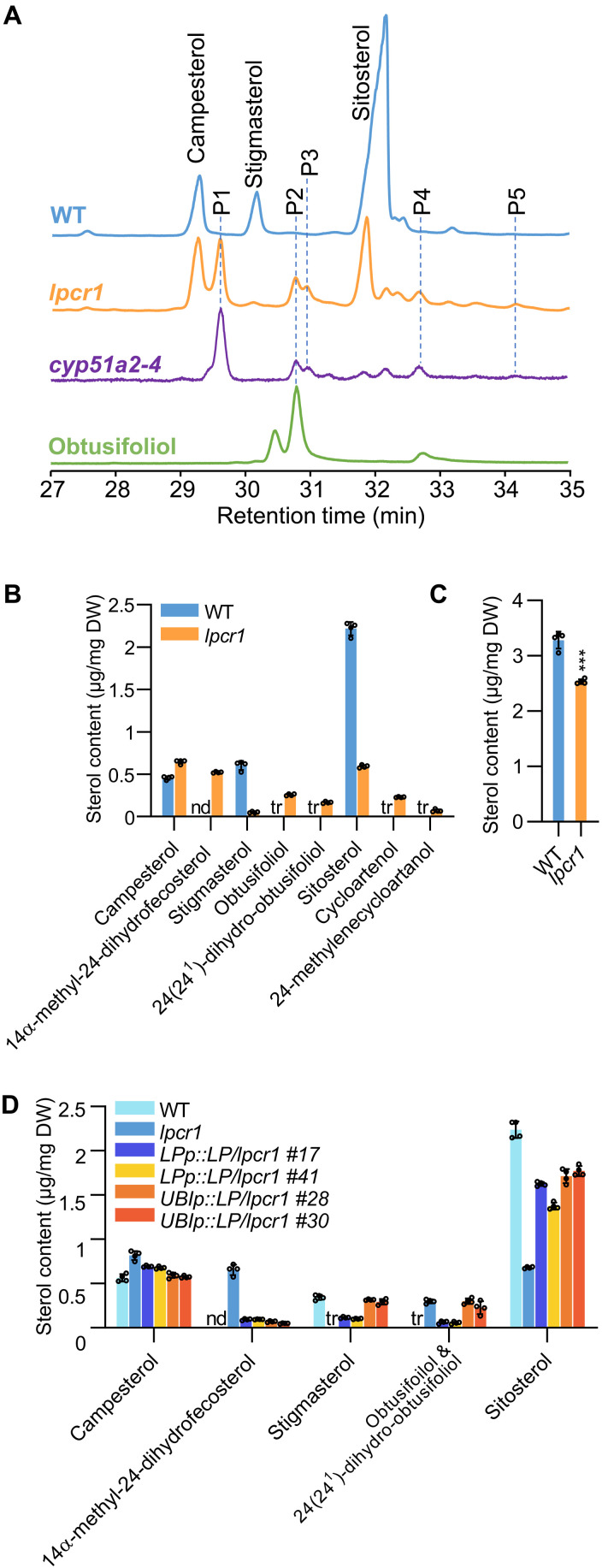
Sterol composition in the *lpcr1* mutant and complementary lines. (**A**) GC-MS total ion chromatograms of sterols extracted from the 10-day-old seedlings of *lpcr1*, *cyp51a2-4*, and WT. Obtusifoliol was included as an authentic standard. P1, 14α-methyl-24-dihydrofecosterol; P2, Obtusifoliol; P3, 24(24^1^)-dihydro-obtusifoliol; P4, Cycloartenol; and P5, 24-methylenecycloartanol. (**B** and **C**) Sterol composition (B) and total sterol content (C) in 10-day-old seedlings of WT and *lpcr1* mutants. (**D**) Sterol composition in 10-day-old seedlings of *lpcr1* complementation lines. nd, not detected; tr, trace amount. Data are presented as means ± SD from four biological replicates. Asterisks denote significant differences with ***P < 0.001 (two-tailed Student’s *t* tests).

We next investigated sterol composition of *CB5LP* mutants. Saponified sterols were extracted from 10-day-old seedlings and then were acetylated and profiled via GC-MS. Unexpectedly, sterol composition in *lpcr1* mutant displayed significant differences, compared to the WT (Col-0). Among the three dominant phytosterols, campesterol, stigmasterol, and sitosterol, that were abundant in the WT seedlings, the levels of stigmasterol and sitosterol were drastically reduced, while five additional steroid metabolites emerged in *lpcr1* seedlings (Fig. 6A). By comparing retention times and mass spectra of acetylated derivatives with those of previously reported steroid species (*38*, *39*), we identified them as 14α-methyl-24-dihydrofecosterol (P1), obtusifoliol (P2), 24(24^1^)-dihydro-obtusifoliol (P3), cycloartenol (P4), and 24-methylenecycloartanol (P5), respectively (Fig. 6A and fig. S9 and S10). The use of available authentic standard further confirmed P2 as obtusifoliol based on its identical retention time and mass spectrum (Fig. 6A and figs. S9I and S10C). All five 14α-methyl-sterols accumulated to high levels in *lpcr1* but were either absent or present only in trace amount in WT (Fig. 6B and fig. S10). Whereas the levels of stigmasterol and sitosterol decreased, campesterol showed a slight increase in *lpcr1* (Fig. 6B), implicating an independent regulation of the 24-ethylidene and 24-methylene sterol pathways. As a result, total sterol content in *lpcr1* was lower than that in WT (Fig. 6C). Similarly, *cb5lp-1* knockdown mutant also accumulated these five sterols, albeit at lower levels than *lpcr1*, and the total sterol content remained unchanged in *cb5lp-1* (fig. S11). Complementation with *CB5LP* in *lpcr1* substantially restored sterol compositions and accumulation levels to those of the WT (Fig. 6D).

To further confirm these findings, we obtained an additional *CB5LP* mutant, a transposon-insertion mutant *pst14737*, which disrupts *CB5LP* in the Nossen-0 background, from the RIKEN BioResource Research Center (fig. S12A). The isolated homozygous line, designated *pst-lp*, exhibited the same seed defects and seedling lethality as *lpcr1*, along with similar sterol composition changes (fig. S12, B to G). These findings, replicated across independent mutant lines, strongly confirm that the loss of CB5LP function results in sterol biosynthesis defects. Therefore, CB5LP is critical for phytosterol biosynthesis.

### CB5LP functions as an electron carrier for CYP51A2 demethylase

The enhanced accumulation of obtusifoliol and its derivatives 24(24^1^)-dihydro-obtusifoliol and 14α-methyl-24-dihydrofecosterol (Fig. 6) in *CB5LP* mutants suggests a disruption in sterol biosynthesis at the step catalyzed by CYP51A2, the *Arabidopsis* obtusifoliol 14α-demethylase (*18*). CYP51A2 was identified as one of the candidate proteins associated with CB5LP through TurboID-based protein proximity labeling analysis (Fig. 5). To further validate their physical interaction or close spatial proximity in planta, we used the pupylation-based interaction tagging (PUP-IT) system (*40*). In this system, CB5LP was fused to the bacterial Pup ligase PafA and served as bait, while CYP51A2-YFP-HA served as prey. These constructs were either expressed individually or coexpressed in *N. benthamiana* (fig. S13A). In samples expressing CYP51A2-YFP–hemagglutinin (HA) alone, a YFP immunosignal, but not FLAG signal, was detected in both input and output fractions, indicating proper expression of the fusion protein without pupylation. In contrast, coexpression of CYP51A2-YFP-HA, FLAG-Pup(E), and CB5LP-PafA resulted in strong immunosignals for both YFP and FLAG, appearing in a characteristic ladder-like pattern in input and output samples (fig. S13B). These results indicate that CYP51A2-YFP-HA underwent pupylation at multiple sites, mediated by the physically associated or proximally located CB5LP-PafA. As a negative control, we tested tCNX1-YFP-HA, a construct containing the C-terminal TM domain of the ER-resident protein CNX1 (tCNX1) (*33*), under the same conditions. This control protein failed to undergo pupylation by CB5LP-PafA (fig. S13C), confirming the specificity of the observed interaction. Together, these results provide in vivo evidence that CB5LP-PafA specifically associates or interacts with CYP51A2-YFP-HA, facilitating its pupylation and supporting a close spatial relationship between the two proteins in planta.

Analysis of isolated homozygous *cyp51a2-4* (SALK_067630) seedlings confirmed the accumulation of 14α-methyl-sterols, including 14α-methyl-24-dihydrofecosterol and obtusifoliol (Fig. 6A), consistent with previous studies on *cyp51a2-3* allele (*18*). Phenotypically, we also observed deformed seeds in the *cyp51a2-4/+* progeny, as well as tiny seedlings that were even smaller than those of the *lpcr1* mutant (fig. S14, A to C). In terms of phenolics metabolism, anthocyanins accumulation was significantly increased in *cyp51a2-4* seedlings, mirroring the observation in *lpcr1*, although its sinapoyl ester and kaempferol levels remained unchanged (fig. S14, D to F). This suggests that *cyp51a2* and *cb5lp* mutant lines may have a similar secondary stress response effect associated with their growth defects. As a cytochrome P450 enzyme, CYP51A2 requires redox partner(s) to supply electrons to support its catalysis. These observations prompted us to investigate whether CB5LP acts as an electron carrier for CYP51A2.

**Fig. 7.**
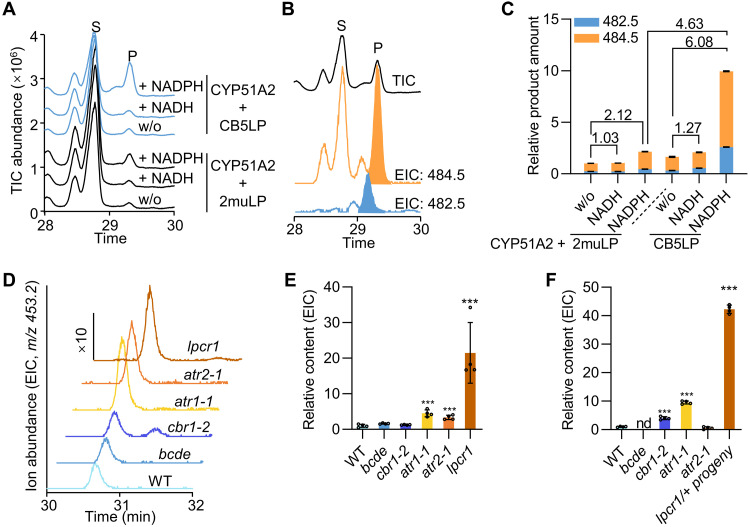
CB5LP functions as an electron shuttle for CYP51A2. (**A**) GC-MS total ion chromatogram (TIC) of CYP51A2-catalyzed reactions. Microsomal proteins prepared from yeast cells coexpressing CYP51A2 with CB5LP or 2muLP were incubated with obtusifoliol, in the presence of NADH or NADPH as reductant. Control reactions were conducted without (w/o) reductant. After a 3-hour reaction at 28°C, reaction products were extracted, derivatized, and resolved by GC-MS. (**B**) Extracted ion chromatogram (EIC) of the reaction of CYP51A2 together with CB5LP in the presence of NADPH, showing the detection of two enzymatic products 4α-methylergostatrienol (*m/z* 482.5) and 4α-methylergostadienol (*m/z* 484.5). S, substrate; P, product. (**C**) Relative product amount of CYP51A2 reaction calculated from the sum of EIC abundance of *m/z* 484.5 and *m/z* 482.5. The product amount in the reaction of CYP51A2 with 2muLP in absence of reductant was set as 1. Data are presented as means ± SD from three technical replicates in one representative experiment. The experiments were repeated three times with similar results. (**D**) EIC detection of obtusifoliol at *m/z* 453.2 in 10-day-old seedlings. Note that scale of EIC for *lpcr1* is 10 time higher than those of the other genotypes. (**E** and **F**) Relative content of obtusifoliol in 10-day-old seedlings (E) and in mature seeds (F) of WT and mutants (*bcde*, *cbr1-2*, *atr1-1*, *atr2-1*, and *lpcr1*). The *lpcr1/+* progeny seeds in (F) represent a mixture of *lpcr1*, *lpcr1/+*, and WT genotypes. Relative obtusifoliol content were calculated on the basis of EIC abundance. The obtusifoliol content in WT was set as 1. Data in (E) and (F) are presented as means ± SD from four biological replicates. Asterisks denote significant differences with ***P < 0.001 (two-tailed Student’s *t* tests). nd, not detected.

We coexpressed CYP51A2 with either AtCB5LP or its mutant variant, 2muLP, in yeast cells. Following the isolation of yeast microsomal proteins, we conducted in vitro CYP51A2 activity assays. When using microsomal proteins from yeasts coexpressing CYP51A2 and 2muLP, we detected low but discernible activity even in the absence of NADH and NADPH (Fig. 7, A to C). This likely reflects background activity facilitated by yeast endogenous electron transfer systems, resulting in minor conversion of obtusifoliol to 4α-methylergostatrienol [mass/charge ratio (*m/z*) of 482.5] and the reduced form, 4α-methylergostadienol (*m/z* of 484.5) (Fig. 7, A to C, and fig. S15). While the addition of NADH barely increased CYP51A2 activity (Fig. 7, A and C), when NADPH used as the cofactor, CYP51A2 activity was significantly enhanced, approximately doubling that detected in the reaction without a reductant. This indicates that NADPH is more effective than NADH in supporting CYP51A2 activity in yeast microsomes (Fig. 7, A and C).

When using microsomes from yeasts coexpressing CYP51A2 and CB5LP, CYP51A2 activity remained low in the presence of NADH (Fig. 7, A and C). However, with NADPH, CYP51A2 activity showed an increase of 4.6-fold compared to that was detected when CYP51A2 was coupled with 2muLP and sixfold compared to that without a reductant or with NADH (Fig. 7C). These results unequivocally demonstrate that CB5LP functions as an electron carrier enhancing CYP51A2 catalysis, with NADPH as a more effective reductant cofactor in supporting CYP51A2 activity when yeast electron transfer chains are supplemented with AtCB5LP.

**Fig. 8.**
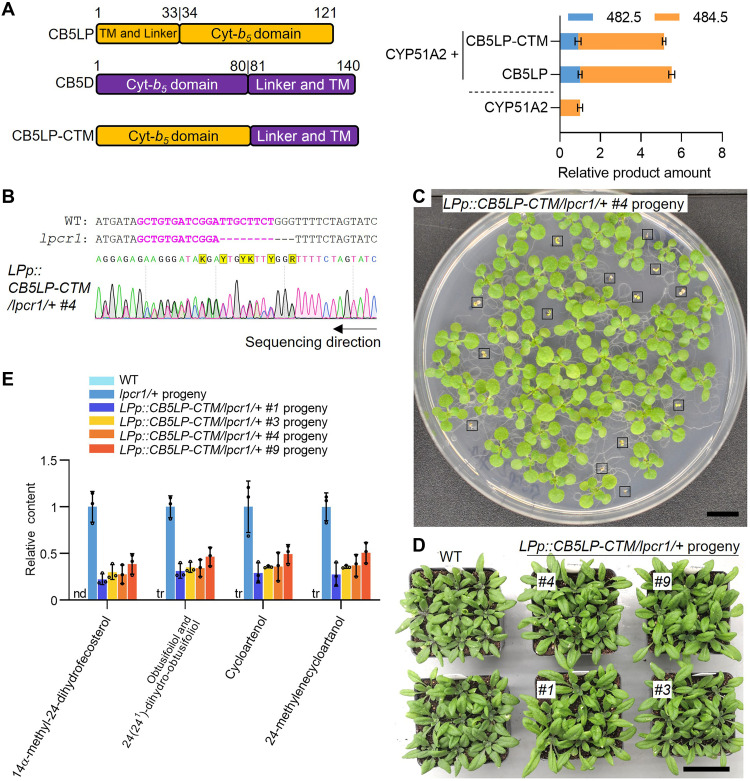
Characterization of domain swapping mutant variant of CB5LP. (**A**) Generation of domain swapping mutant variant CB5LP-CTM and enzymatic assay of CYP51A2 activity. Microsomal proteins were prepared from yeast cells harboring CYP51A2 alone or coexpressing CB5LP or CB5LP-CTM. Reactions were carried out using obtusifoliol as the substrate and NADPH as the reductant. Ion abundances of *m/z* 482.5 and *m/z* 484.5 were calculated to indicate the activity of CYP51A2. The product amount in reaction of CYP51A2 alone was set as 1. Experiments were performed three times independently with similar results. Data are presented as means ± SD of three technical repeats from one representative experiment. (**B**) Sanger sequencing chromatogram confirming the heterozygous *lpcr1/+* genotype in the T1 generation of the representative transgenic line *LPp::CB5LP-CTM/lpcr1/+* #4. (**C**) Morphology of 2-week-old T2 seedlings growing on 1/2 MS medium containing hygromycin, demonstrating successful complementation of the *lpcr1* mutant. Hygromycin-sensitive (nontransgenic) seedlings are boxed. Twelve independent transgenic lines were screened, all showing consistent results. (**D**) Morphology of 5-week-old T2 plants from *LPp::CB5LP-CTM/ lpcr1* transgenic lines, derived from (C), growing in soil. Scale bar, 1 cm (C) and 5 cm (D). (**E**) Relative content of 4α-methyl-24-dihydrofecosterol, Obtusifoliol and 24(24^1^)-dihydro-obtusifoliol, Cycloartenol and 24-methylenecycloartanol in mature seeds of T2 *LPp::CB5LP-CTM/ lpcr1* transgenic lines. Sterol content in *lpcr1/+* progeny seeds was set as 1. Data are presented as means ± SD from three technical replicates. nd, not detected; tr, trace amount.

The primary structural difference of CB5LP relative to the tail-anchored canonical CB5 proteins is its TM domain located at the N terminus (Fig. 8A). To explore the potential functional impact of this structural variation, we generated a CB5LP chimeric variant, CB5LP-CTM, in which the TM domain of the canonical AtCB5D was placed after the cyt-*b_5_* domain of CB5LP (Fig. 8A). In vitro assays revealed that, when coupled with CYP51A2, CB5LP-CTM enhanced CYP51A2 catalytic activity to a level comparable to that of the authentic CB5LP (Fig. 8A), suggesting that the location of TM domain of CB5LP has little effect on its redox function to CYP51A2 in yeast microsome. To assess whether CB5LP-CTM functions in planta, we introduced the *CB5LP-CTM* chimera in a hygromycin-resistant expressing cassette into heterozygous *lpcr1/+* mutants, using the same complementary strategy as in previous assays with *CB5LP* and *2muLP*. Sanger sequencing confirmed the *lpcr1/+* heterozygous background in T1 plants (Fig. 8B). In the T2 generation, all hygromycin-resistant seedlings exhibited growth comparable to the WT, with no lethality observed (Fig. 8, C and D). The presence of the *lpcr1* allele and the homozygous *lpcr1* background in selected T2 individuals were confirmed by genomic PCR and Sanger sequencing (fig. S16). Furthermore, the accumulation of 14α-methyl-sterols, which was prominent in *lpcr1/+* progeny seeds, was substantially reduced in the *LPp::CB5LP-CTM/lpcr1/+* progeny seeds (Fig. 8E). These findings confirm that CB5LP-CTM effectively complements *lpcr1* deficiency. Together, our results confirm that CB5LP functions as an electron shuttle for CYP51A2, with its functionality and specificity determined by its cyt-*b_5_* domain but is independent of the location of its TM domain.

### CPR and CBR function as tissue-specific redox components for CYP51A2

To further investigate the electron transfer chain(s) involved in CYP51A2 catalysis, we detected accumulation of obtusifoliol, the substrate of CYP51A2, in the mutants deficient in other ER electron transfer components, including *cbr1-2*, *atr1-1*, *atr2-1*, and the canonical *CB5* quadruple mutant *bcde* (*34*), with *lpcr1* as a control. Notably, obtusifoliol levels remained unchanged in seedlings of *bcde* mutants but decreased in their seeds, suggesting that the four ER-localized canonical CB5s likely do not contribute to CYP51A2 function in seedlings and even suppress its function in seeds (Fig. 7, D to F). A tissue-specific accumulation of obtusifoliol was observed in *cbr1-2*, *atr1-1*, or *atr2-1* mutant. Obtusifoliol levels increased in the seedlings of *atr1-1* and *atr2-1* mutants but remained unchanged in *cbr1-2* (Fig. 7, D and E); in contrast, obtusifoliol accumulated in the seeds of *cbr1-2* and *atr1-1* mutants but not in *atr2-1* (Fig. 7F). These results suggest that CBR and CPR isoforms, in cooperation with CB5LP, can serve as electron donors to CYP51A2 for sterol biosynthesis, but they function in a tissue-specific manner. Specifically, CBR1 and ATR1 function in seeds, while ATR1 and ATR2 act in seedlings. This tissue-specific electron transfer pattern highlights a complex regulatory mechanism controlling CYP51A2 activity across different plant tissues.

### CB5LP homologs exclusively present in plant kingdom

Given the topological difference between CB5LP and canonical tail–anchored CB5 proteins, we conducted a comprehensive search for CB5LP and canonical CB5 homologs across the domains of life (data S5 to S8). All the hits were then examined for the presence of cyt-*b_5_* domain using the HMMER algorithm (http://hmmer.org/) and predicted TM helices using TMHMM algorithm (*41*). All the hits obtained from green plants and red algae were subjected to phylogenetic analysis, while the hits from Bacteria, Fungi, and Metazoa were retained on the basis of the following criteria: peptide lengths between 100 and 200 amino acids, start codon as methionine, cyt-*b_5_* domain of at least 70 amino acids, and containing one or no TM helix.

We found CB5LP homologs in all plant species, spanning diverse evolutionary lineages, including Red algae (*Porphyridium purpureum*, *Gracilaria domingensis*, *Rhodosorus marinus*, *Cyanidium caldarium*, *Gracilariopsis chorda*, and *Cyanidioschyzon merolae*), Chlorophyte algae (*Chlamydomonas reinhardtii* and *Volvox carteri*), Charophyceae algae (*Chara vulgaris)*, Bryophytes (*Marchantia polymorpha* and *Physcomitrium patens*), Lycophytes (*Selaginella moellendorffii*), Monilophytes (ferns) (*Salvinia cucullata* and *Ceratopteris richardii*), Gymnosperms (*Pinus radiata*, *Ginkgo biloba*), and Angiosperms (*Amborella trichopoda*, *Oryza sativa*, *Populus trichocarpa*, and *Petunia axillaris*) (data S5). In contrast, the 329 fungal hits and 415 metazoan hits, obtained using either AtCB5LP or AtCB5D as query, all retained a single TM domain at their C terminus, a hallmark of the tail-anchored canonical CB5 proteins (data S6 and S7). This strongly indicates that CB5LP homologs are absent from fungi and animals.

Among the identified 544 bacterial hits, 388 contained a single TM helix, while 156 lacked a TM domain (data S8). Notably, 387 of the 388 single TM proteins exhibited an N-terminal TM domain, a defining characteristic of CB5LP protein. The only exception, WP_318072142.1, retained C-terminal TM. However, further examination suggested that it is likely an environmental contaminant. Especially, when WP_318072142.1 was used as a query for BLSTP search against nonredundant protein sequences in GenBank, the top 99 hits were canonical CB5 proteins from Eutheria (data S9), inferring a possible sequence contamination from placental mammals. Furthermore, when canonical AtCB5D was used as a query against bacterial sequences, except WP_318072142.1, all 443 retrieved hits retained an N-terminal TM domain, confirming that bacterial CB5 homologs are structurally CB5LP-like (data S10). These data indicate that the TM-containing cytochrome *b_5_* proteins in bacteria predominantly adopt CB5LP-type topology; therefore, CB5LP represents an ancestral isoform of cytochrome *b_5_* proteins.

**Fig. 9.**
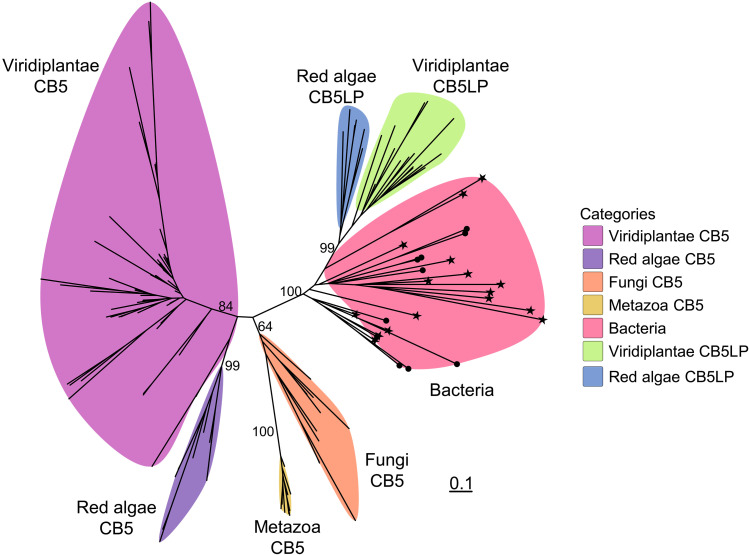
Phylogeny of CB5 and CB5LP homologs from representative taxa of all life domains. CB5 and CB5LP homologs of Viridiplantae and Red algae were obtained using AtCB5D and AtCB5LP as queries via BLASTP search against Phytozome (*C. reinhardtii*, *V. carteri*, *M. polymorpha*, *P. patens*, *S. moellendorffii*, *C. richardii*, *A. trichopoda*, *O. sativa*, and *P. trichocarpa*), FernBase (*S. cucullata*), Genbank (*P. purpureum*, *G. domingensis*, *R. marinus*, *C. caldarium*, *G. chorda*, and *C. merolae*), ONEKP (*C. vulgaris*, *P. radiata*, and *G. biloba*), and Solanaceae Genomics Network (*P. axillaris*). Metazoa, Fungi, and Bacteria CB5 and CB5LP homologs were obtained using AtCB5LP as query via BLASTP search against Clustered nr (nr_clustered) database. Maximum likelihood tree was constructed with full-length proteins using IQ-tree2 algorithm (Q.pfam+R6 model selected by ModelFinder, 1000 SH-aLRT tests, and 1000 ultrafast bootstraps). Only major bifurcate bootstrap support values are marked on the main branches. Bacterial proteins with and without a single TM domain are symbolized with stars and dots, respectively. The scale bar indicates average number of amino acid substitutions per site.

To explore the evolutionary relationships of CB5 and CB5LP, we constructed phylogenetic trees using full-length peptide sequences of all CB5 and CB5LP homologs from representative plant species and the top hits from bacteria, fungi, and metazoans, and by using cyt-*b_5_* domain sequences extracted using the HMMER algorithm (Fig. 9 and fig. S17 and S18). Both phylogenies exhibited similar topologies, indicating that the cyt-*b_5_* domain is indicative of the evolutionary relationship of CB5 family proteins. Within constructed phylogeny, bacterial, fungal, plant, and animal proteins clustered into separated, distinct clades, reflecting their kingdom-specific divergence. Bacterial homologs, regardless of whether they had a predicted TM domain or not, are clustered into a single clade. Further examination of the 156 bacterial proteins predicted without a TM domain revealed that many of them contain TM domain–like sequences, implying a potential misannotation of their sequences (data S11). For the CB5 sequences identified from Viridiplantae and red algae, they clearly clustered into distinct canonical CB5 and CB5LP clades, indicating their different evolutionary origins. CB5LP homologs from red algae and Viridiplantae clustered closely with bacterial CB5 homologs, together forming a monophyletic group. This strongly suggests that plant CB5LPs may arise from a bacterial ancestor likely via horizontal gene transfer (HGT) or endosymbiosis (Fig. 9). Unlike plants, fungi and animals lack CB5LP homologs, and their CB5 proteins clustered close to the plant canonical CB5 clade, suggesting that the tail-anchored CB5 proteins in eukaryotes evolved from a common ancestor that might be distinct from the one evolving to plant CB5LPs. These findings support the hypothesis that CB5LP represents an ancient, bacterial-derived cytochrome *b_5_* variant, which was likely recruited into plants via HGT or endosymbiosis and subsequently evolved into a specialized electron donor for phytosterol biosynthesis.

Subsequently, we synthesized 10 bacterial genes encoding CB5LP-like proteins with a single N-terminal TM domain and coexpressed them with *AtCYP51A2* in yeast to assess their potential effects on CYP51A2 activity (data S12 and fig. S19A). None of these bacterial CB5LP-like protein enhanced the demethylase activity of CYP51A2 (fig. S19A). However, when three of them, as representatives, were processed for recombinant protein production to determine whether they convey electron transfer capability, we found that the purified recombinant proteins with removal of their TM domains exhibited typical heme-bound CB5 protein properties, e.g., showing an absorption maximum at 413 nm. When incubated with *Arabidopsis* CBR1 in the presence of NADH or ATR2 in the presence of NADPH, these proteins were efficiently reduced, showing absorptive shift to the prominent peaks at 424, 526, and 557 nm, characteristic of reduced CB5 proteins (fig. S19, B to G). These results indicate that bacterial CB5 homologs are the prototypes of CB5LP proteins that have electron transfer properties. These proteins, after being recruited into plants, may have undergone functional specialization, including their evolving capacity to support CYP51A2-catalyzed sterol demethylation.

## DISCUSSION

Phytosterols are essential components of plant cell membranes, regulating their fluidity and permeability. In addition, they can regulate the activity and distribution of integral membrane proteins, including enzymes, ion channels, and signal transduction components (*42*). Chemically, phytosterols serve as precursors for bioactive molecules, including brassinosteroid hormones, which regulate plant developmental processes (*43*), and the synthesis of a variety of secondary metabolites (*42*).

In this study, we identified CB5LP, a unique cytochrome *b_5_* family member, as an essential component of phytosterol biosynthesis. Genetic and biochemical analyses reveal that CB5LP acts as an electron carrier supporting sterol 14α-demethylase (CYP51) activity. Null mutants of *CB5LP* (*lpcr1* and *pst-lp*) accumulated higher levels of 14α-methyl-sterols, including 14α-methyl-24-dihydrofecosterol, and obtusifoliol, the authentic CYP51 substrate (Fig. 6B and fig. S12G). This accumulation closely resembles that observed in the *Arabidopsis cyp51a2-3* mutant (*18*) and *Nicotiana* plants in which *CYP51* is silenced (*44*). Moreover, *CB5LP* disruption caused embryonic defects and seedling lethality, mirroring the phenotypes of severe *CYP51A2* allelic mutant *cyp51a2-4* (Fig. 2 and fig. S14) and *cyp51a2-3* (*18*). Although topologically distinct from canonical tail–anchored CB5 proteins, both in vitro redox assays and in planta validation confirm that CB5LP functions as an ER-localized electron carrier protein (Fig. 4). Its two histidine residues, predicted to bind a heme molecule, are essential for its redox functioning (Fig. 4), like the canonical CB5 proteins (*30*, *45*). In vitro assays further confirmed that when CB5LP coexpresses with AtCYP51A2, it substantially enhanced the latter’s 14α-demethylase activity in yeast microsomes, whereas the CB5LP inactive variant 2muLP failed to do so (Fig. 7, A to C). These results confirm that CB5LP functions as an electron donor, by associating with CYP51, regulating phytosterol biosynthesis. In addition, the absence of obtusifuliol accumulation in the high-order *bcde* mutant, which is deficient in four ER-resident canonical *CB5* genes, further supports the unique and essential role of CB5LP in supporting CYP51A2 catalysis (Fig. 7, D to F).

The disruption of *CB5LP* and *CYP51A2* resulted in differential effects on the production of the primary phytosterol end products in *Arabidopsis*, although their deficiency yielded in the similar accumulation of biosynthetic intermediate obtusifoliol and the related derivatives (Fig. 6). The loss of CYP51A2 activity substantially impaired the production of all three primary phytosterols, i.e., campesterol, sitosterol, and stigmasterol, whereas deficiency of CB5LP predominately reduced the accumulation of sitosterol and stigmasterol but slightly enhanced the level of campesterol (Fig. 6). These results suggest that the biosynthesis of different types of phytosterols is not achieved through a linear pathway. The route to 24-ethylidene sterols including sitosterol and stigmasterol might be independent from the route to 24-methylene sterols leading to campesterol and/or brassinosterols, and CB5LP might be selectively recruited by the CYP51 and involved in the regulation of 24-ethylidene sterol biosynthesis. In addition, the CB5LP proxiome revealed CYP710A2, a sterol C22 desaturase responsible for the conversion of sitosterol to stigmasterol, as a potential associated partner (data S2). Disruption of *CB5LP* appeared to more severely impair accumulation of stigmasterol than sitosterol, implicating that CB5LP may also directly support CYP710A2 activity (Fig. 6), although their redox partnership remains to be biochemically validated. In contrast, none of the cytochrome P450 enzymes involved in campesterol-derived brassinosteroid biosynthesis (such as CYP90A1, CYP90B1, CYP90C1, CYP85A1, and CYP85A2) (*46*) were identified as potential CB5LP interactors (data S2). These data further support the functional specialization of CB5LP in 24-ethylidene sterol biosynthesis rather than in the 24-methylene sterol–related brassinosteroid pathway and an independent regulation of phytosterol branch biosynthetic pathways.

Furthermore, disruption of CYP51A2 led to a nearly complete loss of the three main phytosterols, whereas the loss of CB5LP resulted only in the partial reduction of 24-ethylidene sterol accumulation (Fig. 6). These findings suggest that, in addition to CB5LP, other electron donors may contribute to CYP51A2-mediated catalysis.

Also of note is that although CB5LP exhibited a kinetic preference for its cognate reductase NADH-dependent CBR1 over NADHP-dependent ATR1 and ATR2, yeast microsome–based enzymatic activity assays revealed a preference of CPY51A2-CB5LP for NADPH over NADH in supporting obtusifoliol 14α-demethylation (Fig. 7C). In seedlings, the defect of *ATR1* or *ATR2* but not *CBR1* resulted in the accumulation of obtusifoliol, whereas in seeds, *ATR1* or *CBR1* deficiency led to obtusifoliol accumulation (Fig. 7, E and F). These data suggest that *Arabidopsis* CYP51A2 uses both the NADPH-dependent ATR-CB5LP and the NADH-dependent CBR-CB5LP electron transfer pathways for its catalysis in different tissues, in which CB5LP acts as a central electron carrier. A similar dual electron transport mechanism was adopted by the relatively recently evolved P450 F5H that is responsible for angiosperm syringyl lignin biosynthesis (*34*). Expectedly, adopting such dual electron transport systems provides necessary flexibility and versatility for P450 enzymes, allowing them to efficiently use available reductants under varying cellular conditions. In our previous study, we observed expression of *ATR1*, *ATR2*, and *CBR1* in examined tissues including stem, young and old leaves, flowers, siliques, and developing seeds. Notably, ATR1 exhibited relatively higher expression in 14-day-old leaves compared to developing seeds, whereas CBR1 showed higher expression in 6-day-old developing seeds than in 14-day-old leaves (*34*). However, the molecular basis for how ER-localized electron transfer chains are selectively recruited in different tissues to support specific P450 enzymes remains to be clarified.

Sterol 14α-demethylase (CYP51) is the only P450 enzyme required for sterol biosynthesis across different phyla. It is the most widely distributed P450 family, present in animals, plants, fungi, and even some bacteria such as *Mycobacterium tuberculosis* and *Methylococcus capsulatus* (*15*, *47*, *48*). In *M. capsulatus*, sterol biosynthesis follows an abbreviated pathway, where its soluble CYP51 enzyme is fused to a ferredoxin domain at its C terminus, allowing it to receive necessary reducing equivalents directly (*48*). In contrast, eukaryotic membrane–bound CYP51s require sequential electron input from redox partners to complete their monooxygenation cycle. Previous studies revealed that yeast and human CYP51 catalysis primarily relies on CPR as its electron donor, while canonical tail–anchored CB5 protein can play a supplementary role (*49*). However, depletion of *CB5* gene in yeast did not cause lethality or affect growth unless the endogenous *CPR* gene was also disrupted, suggesting that CB5 is nonessential for yeast or animal CPY51 function (*49*–*51*). In notable contrast, the loss of *CB5LP* in *Arabidopsis* led to embryonic defects and seedling lethality, the phenotype comparable to the loss of *CYP51A2* (*18*). This suggests that CB5LP is necessarily required for plant CYP51 activity and plant development. This unique redox partnership distinguishes plant sterol biosynthetic P450 from those in other eukaryotes, presenting a potential opportunity by targeting CYP51 electron donors to develop specific herbicides for agricultural applications.

Phylogenetic analysis suggests that CB5LP originated from bacterial prototype CB5 proteins and is uniquely present in plant lineages, including red algae and green species from the unicellular green algae *C. reinhardtii* to flowering plants such as *Arabidopsis*, *Populus*, and rice (Fig. 9 and fig. S17). Unlike canonical CB5 proteins, which have undergone substantial expansion and diversification during land plant evolution (*52*), CB5LP has retained a single member across all plant taxa examined to date. This pattern suggests an essential housekeeping role in plant growth, development, and metabolism. A similar evolutionary conservation is observed for the sterol biosynthetic CYP51 enzymes, which are also highly conserved across plant lineages (*53*). Given the sequence and/or functional conservation of both CYP51A and CB5LP across diverse plant species (fig. S18) (*53*), it is likely that the unique redox partnership between CB5LP and CYP51A and the electron carrier function of CB5LP in supporting CYP51-catalyzed phytosterol biosynthesis are conserved throughout the plant kingdom. Further investigation into the CB5LP-CYP51 functional interaction in unicellular green algae and other land plants could provide a broader evolutionary perspective and deeper functional insight into this specialized redox partnership in plant sterol biosynthesis.

## MATERIALS AND METHODS

### Plant materials and growth conditions

The *Arabidopsis thaliana* mutant lines *cbr1-2* (Sail_644_A11), *atr1-1* (SALK_208483), *atr2-1* (SALK_152766), and *bcde* were previously described (*34*). The *Arabidopsis* mutant lines *cb5lp-1* (SALKseq_042107), *cyp51a2-4* (SALK_067630), and Nossen-0 (PST-WT, CS1394) were obtained from ABRC, while *pst-lp* (pst14737) was ordered from RIKEN BioResource Research Center. Homozygous T-DNA insertion mutants were identified through genomic DNA genotyping. The *lpcr* mutants were generated using the CRISPR-Cas9 method, following the protocol described by Wang *et al.* (*54*). To isolate T-DNA–free *lpcr1/+* mutants, F2 plants derived from a backcross between *lpcr1/+* and WT were screened by PCR genotyping to identify lines lacking the Cas9 transgene, and by Sanger sequencing to confirm heterozygous *lpcr1/+* genotype. The verified Cas9-free *lpcr1/+* plants were grown to maturity, and F3 seeds were harvested. The absence of the T-DNA was further confirmed by germinating F3 seeds on 1/2 MS medium containing hygromycin, where T-DNA–free seedlings exhibited hygromycin sensitivity. The primers used in genotyping are listed in table S1. For seed germination, the seeds were sterilized with 70% alcohol and sown on 1/2 MS (Phytotech, M524) supplemented with 1% agar and 1% sucrose. After 3 days of stratification at 4°C, the seeds were germinated and maintained at 22°C under a 16-hour light/8-hour dark regime in a BioChambers growth chamber with a light intensity of 8500 lumens/m^2^. The 7-day-old seedlings were then transferred to soil and grown to maturity under the same conditions.

### Generation of transgenic plants

The *pMDC32-UBIp::GW* and *pMDC32-LPp::GW* vectors were constructed on the basis of *pMDC32-35Sp::GW*, in which the *CaMV 35S* promoter was replaced by the *Arabidopsis UBIQUITIN-10* promoter (*UBIp*) and *CB5LP* promoter (*LPp*) at restriction sites of *HindIII*/*KpnI*. The coding region of *CB5LP* gene and the sequence of *LPp* were amplified by PCR using the gene-specific primers, separately. The *2muLP* fragment was generated via overlapping PCR with site-directed mutagenesis primers. The *CB5LP-CTM* fragments were obtained through overlapping PCR. The primer sequences used for PCR amplification are listed in table S1. The *CB5LP*, *LPp*, *2muLP*, and *CB5LP-CTM* were cloned to the gateway cloning entry vector *pDONR207* (Thermo Fisher Scientific) and sequenced, thereby generating entry clones. Subsequently, *pMDC32-UBIp::CB5LP*, *pMDC32-LPp::CB5LP*, *pMDC32-LPp::2muLP*, *pMDC163-LPp::GUS*, and *pMDC32-LPp::CB5LP-CTM* vectors were obtained via Gateway cloning strategy (Thermo Fisher Scientific). The resulting constructs were transferred into *Agrobacterium tumefaciens* strain GV3101 and transformed into *Arabidopsis* with the corresponding backgrounds via the floral dip method (*55*).

Primary transformants were screened on 1/2 MS medium supplemented with hygromycin B (25 mg liter^−1^; Gold Biotechnology). After 2 weeks, the positive seedlings were transplanted into soil and cultivated to maturity in a growth chamber under the previously specified conditions. For the *lpcr1* complementation assay, T-DNA–free *lpcr1/+* individuals were used as the transformation background. In the T1 generation, transformants were PCR-genotyped to confirm the *lpcr1/+* background. Sanger sequencing was then performed to identify homozygous *lpcr1* individuals among the *pMDC32-LPp::CB5LP* transformants and to confirm heterozygous *lpcr1/+* backgrounds for the *pMDC32-LPp::2muLP* and *pMDC32-LPp::CB5LP-CTM* transformants. In the T2 generation, seeds were germinated on 1/2 MS medium containing hygromycin B to assess segregation phenotypes, and Sanger sequencing was used to confirm the *lpcr1* background in hygromycin-resistant, *lpcr1-type* seedlings.

### RT-qPCR and RT-PCR analysis of gene expression

Total RNA was extracted using TRIzol reagent (Thermo Fisher Scientific) following the manufacturer’s guidelines. Reverse transcription of the extracted RNA (1 μg) was conducted using 4 μl of iScript Reverse Transcription Supermix (Bio-Rad) in a 20 μl reaction. The thermal cycler was programmed for incubation at 25°C for 5 min, followed by 46°C for 20 min, and a final step at 95°C for 1 min. The resulting cDNA solution was diluted fivefold, and 2 μl of this dilution was used as a template in a 20-μl PCR reaction with Taq Master Mix (Bio-Rad), or 15-μl qPCR reaction with SsoAdvanced Universal SYBR Green Supermix (Bio-Rad). The qPCR was executed on a CFX96 Real-Time System (Bio-Rad), and the cycle threshold values were determined using the CFX Manager Software v.3.3 (Bio-Rad). The primer sequences used for qPCR and PCR analysis are listed in table S1. *PP2A* and *Actin 2* genes in *Arabidopsis* were used as the housekeeping reference for RT-qPCR and RT-PCR. For relative quantification, qPCR data were analyzed using the delta-delta cycle threshold (ΔΔCt) method (*56*).

For absolute quantification of *CB5LP* expression levels, *CB5LP* transcript copy numbers per unit weight of total RNA were calculated with a derived formula. Serial dilutions (from 10^−1^ to 10^−8^) of pure *pDONR207-CB5LP* plasmid DNA were used to establish a standard curve, with the transcript copy numbers calculated on the basis of the plasmid’s known length. The eight plasmid DNA dilutions, along with cDNA samples from various *Arabidopsis* tissues, were used as templates for qPCR to obtain the corresponding cycle threshold values. The equation: cycle threshold value = −3.1953 × log_10_ (*CB5LP* copy numbers) + 36.599, (with *R*^2^ = 0.9987), was generated using the eight plasmid DNA dilutions. The *CB5LP* transcript copy numbers in total RNA were then determined using this formula.

### CB5LP subcellular localization, membrane fractionation, and immunoblotting

The *CB5LP* gene was subcloned to *pEarleyGate 101* vector, resulting in *35Sp::CB5LP-YFP* construct. *SP-GFP-HDEL* was used as ER marker (*33*). *A. tumefaciens* strain GV3101 carrying the constructs were infiltrated to *N. benthamiana* leaves. After 3 days of incubation, fluorescence images were captured using a TCS SP5 laser-scanning confocal microscope (Leica) with excitation wavelength at 514 nm and an emission wavelength of 520 to 535 nm for YFP signals. Chloroplast autofluorescence was observed at 636 to 725 nm. Membrane preparation was carried out as previously described (*57*). Immunoblotting was conducted with anti-GFP (Thermo Fisher Scientific, A6455) and anti-actin (Sigma-Aldrich, A0480) antibodies following standard gel blot procedures.

### GUS staining

Histochemical analysis of GUS reporter enzyme activity was performed as previously described (*58*, *59*). Briefly, tissue samples from transgenic plants were first incubated in ice-cold 90% acetone at room temperature for 30 min. The samples were then transferred to GUS staining buffer and incubated for a duration at 37°C for 1 to 12 hours. After staining, pigments were removed using 70% ethanol and GUS staining patterns were documented using either a digital camera (Nikon DC3000) or a stereo microscope (Leica M2205 FCA).

### Sterol quantification

Sterol analysis was conducted following a modified protocol based on previous studies (*60*, *61*). Approximately 5 mg of lyophilized 10-day-old seedlings or dried mature seeds were weighed into a glass vial. Saponification was carried out by incubating the plant material in 2 ml of ethanol containing 5 μg of 5-α-cholestane (as internal standard), 1 mg of butylated hydroxytoluene, and 200 μl of 11 N KOH at 90°C for 1 hour with intermittent agitation. After cooling, 1 ml of 0.9% NaCl and 3 ml of hexane were added successively. The vials were vortexed for 1 min and centrifuged for phase-separation. The upper organic phase was collected and vacuum dried. The dried residue was acetylated at 70°C for 90 min with a mixture of 25 μl of pyridine and 25 μl of acetic anhydride. After cooling and vacuum drying, 100 μl of hexane was added and mixed thoroughly. The reaction mixture was then incubated on ice for 5 min followed by centrifugation at 20,000*g* (Eppendorf 5424R centrifuge; FA-45-24-11 rotor). The upper phase was collected for analysis. A 5-μl aliquot was injected to an Agilent 7890A GC-MS and resolved with HP-5MS fused silica capillary column (30 m by 0.25 mm, 0.25-μm film thickness, Agilent). The inlet temperature was set at 300°C and pressure of 19.417 psi. The carrier gas (helium) flow rate was 1 ml/min. The oven temperature program was as follows: 200°C (held for 1 min), ramped to 270°C at 4°C/min (held for 1 min), and then ramped to 290°C at 1°C/min (held for 0.5 min). Detection was performed with a quadrupole spectrometer operating in the electron impact mode with a source temperature of 230°C and quadrupole temperature of 150°C. Sterol composition quantification was expressed either as molar percentage or absolute values. For absolute quantification, a Relative Response Factor (*RRF*) of 0.94 was applied, calculated using the following equation

$$\mathrm{RRF}=\frac{\text{Peak area}\;\left(\text{sitosterol}\right)/\text{mass}\;\left(\text{sitosterol}\right)}{\text{Peak area}\;\left(5-\alpha -\text{cholestane}\right)/\text{mass}\;\left(5-\alpha -\text{cholestane}\right)}$$

### Soluble phenolics analysis

Soluble phenolics composition was analyzed following the established protocol (*34*). Briefly, approximately 50 mg of plant materials were weighed and transferred into a 1.5-ml centrifuge tube. Subsequently, to each tube, 300 μl of 80% (v/v) methanol containing 80 μM chrysin (internal standard) and two steel balls were added. The samples were ball-milled for 2 min at 30 Hz using a CryoMill (Retsch). Following milling, an additional 200 μl of 80% (v/v) methanol containing 80 μM chrysin was added. The samples were then incubated at 4°C for 4 hours. After incubation, 5 μl of the supernatant was injected into either an ultrahigh-performance liquid chromatography (UHPLC) system (Thermo Fisher Scientific) or an HPLC system (Agilent) using the same settings as previously described (*34*).

### Anthocyanin analysis

Anthocyanin content was analyzed following a previously established protocol (*62*). Briefly, plant samples were weighed and transferred into 1.5-ml centrifuge tubes. Then, 45% (v/v) methanol containing 5% acetic acid was added. After homogenization, the samples were centrifuged at 15,000*g* for 5 min at room temperature. The supernatants were transferred to new tubes and centrifuged again under the same conditions. Absorbance at 530 and 657 nm was measured using a Spark microplate reader (Tecan, Männedorf, Switzerland). Relative anthocyanin content was calculated as [Abs_530_-(0.25 × Abs_657_)].

### Plant lipids analysis

Fatty acid methyl esters were prepared following a modified protocol based on a previous report (*63*). For seedling samples, total lipids were extracted from about 100 mg of freshly harvested plants by the addition of 700 μl of methanol: chloroform: formic acid (2:1:0.1, v/v) containing 50 μg of heptadecanoic acid (as internal standard). The samples were vigorously shaken for 2 hours. After adding and mixing 350 μl of 1 M KCl and 0.2 M H_3_PO_4_, the samples were centrifuged at maximum speed. The lower phase–containing total lipids was collected into new tubes. For seeds samples, the whole seed–catalyzed transmethylation protocol was followed.

Transmethylation was performed on 20 μl of total lipids (from seedlings), or 20 seeds. One milliliter of freshly prepared reaction mixture (5% H_2_SO_4_ in methanol, v/v, containing 20 μg of heptadecanoic acid and 50 μg of butylated hydroxytoluene) and 300 μl of toluene cosolvent were added. The samples were heated at 90°C for 1.5 hours. After cooling, 1 ml of 0.9% NaCl and 1 ml of hexane were added successively. The tubes were vortexed for 1 min, phase-separated by centrifugation, and the upper organic phase was collected and vacuum dried. The extracted residuals were redissolved in 100 μl of hexane, and 1 μl of the sample was injected into an Agilent 7890A GC system equipped with an Agilent 60 m DB23 capillary column (inside diameter, 250 μm), and a 5975C mass-selective detector operating in full-mass scan mode. The inlet temperature was set at 250°C and pressure at 12.93 psi. The oven temperature was ramped from 100° to 240°C at a rate of 15°C min^−1^ and held at 240°C for 3 min with a carry gas flow rate of 1.2 ml min^−1^.

For TLC analysis, total lipids were isolated from leaves following the procedure described above, except that the internal standard was excluded. Lipid extracts were separated on silica plates (Silica Gel 60, Sigma-Aldrich) by using a solvent system consisting of hexane/diethyl ether/acetic acid (70:30:1, v/v/v) (*64*). Lipids on TLC plates were visualized by spraying with 0.05% primuline in acetone/water (80:20, v/v). After drying, plates were viewed under an ultraviolet (UV) lamp, and images were captured.

### Glucosinolates analysis

Glucosinolates extraction and desulfoglucosinolate preparation were prepared following a modified protocol based on previous reports (*65*, *66*). Two-week-old *Arabidopsis* seedlings grown on 1/2 MS medium were harvested, lyophilized, and ground into a fine powder. A total of 10 mg of plant material was weighed into 2-ml round-bottom screw-cap tubes. To each tube, 1 ml of 70% methanol was added, followed by 5 μl of 10 mM sinigrin solution (Sigma-Aldrich, 85440) as an internal standard. The mixture was heated in a water bath at 90°C for 5 min and then subjected to ultrasonic treatment for 15 min. The tubes were centrifuged at 20,000*g* for 5 min, and the supernatant was transferred to a new tube. The residue was reextracted by adding another 1 ml of 70% methanol, repeating the heating, ultrasonic treatment, and centrifugation steps. The two supernatants were combined and centrifuged again at 20,000*g* for 5 min.

The final supernatant was transferred to a microcentrifuge tube containing 200 μl of Sephadex A-25 resin (Sigma-Aldrich, A25120). The mixture was inverted several times for thorough mixing and then centrifuged at 1000 rpm for 1 min. The resin was sequentially washed with 70% methanol (twice), water (once), and 20 mM sodium acetate (twice). Subsequently, 5 μl of sulfatase (Sigma-Aldrich, S9626) and 45 μl of 20 mM sodium acetate were added to the resin, and the sample was incubated overnight at 37°C. After incubation, 70 μl of water was added to elute the desulfoglucosinolates.

For UHPLC-MS analysis, 5 μl of the eluted sample was injected into a UHPLC system coupled with a Q Exactive Plus mass spectrometry system (Thermo Fisher Scientific). Separation was achieved on a reverse-phase C18 column (Luna, 150 by 2.1 mm, 1.6 μm; Phenomenex). The gradient program was as follows: 2% B (0 min); 40% B (25 min); 99% B (26 min); 99% B (33 min); 2% B (34 min); 2% B (35 min) at a flow rate of 0.1 ml/min. The mobile phases A and B were 0.1% acetic acid in water and 0.1% acetic acid in acetonitrile, respectively. Compounds were detected using an Ultraviolet-Visible (UV-Vis) diode array detector at 229 nm. The mass spectrometer was operated with parameters as described earlier. Mass spectrometry data were used to identify 4MSOB (*m/z* 194), sinigrin (*m/z* 116), 5-methylsulfinylpropyl (*m/z* 208), 4MTB (*m/z* 178), 8-methylsulfinyloctyl (*m/z* 250), indol-3-ylmethyl (*m/z* 205), 4-methoxyindol-3-ylmethyl (*m/z* 235), and 1-methoxyindol-3-ylmethyl (*m/z* 235, MS2: 205).

### TurboID assay

The *CB5LP* and *mGFP5* sequences were PCR-amplified from the *pDONR207-CB5LP* and *pEarleyGate 103* plasmids, respectively. These fragments were inserted into the *pMDC32-UBIp::GW* vector at *Kpn I* and *Spe I* restriction sites to generate the *pMDC32-UBIp::CB5LP-mGFP5* construct. Subsequently, *CB5LP-mGFP5* and *TurboID* fragments were PCR-amplified from *pMDC32-UBIp::CB5LP-mGFP5* and *R4pGWB601_UBQ10p-Turbo-NES-YFP* (Addgene, plasmid #127366), respectively. These fragments were inserted into *pMDC32-UBIp::GW* vector at *Kpn I* and *Spe I s*ites to create the *pMDC32-UBIp::CB5LP-mGFP5-TurboID* construct. In addition, *TurboID* sequence was PCR-amplified and inserted into *pMDC32-UBIp::GW* vector at *Kpn I* site to generate the *pMDC32-UBIp::TurboID-GW* vector. An LR reaction was then performed to produce the *pMDC32-UBIp::TurboID-mGFP5* construct.

The obtained *pMDC32-UBIp::CB5LP-mGFP5-TurboID* and *pMDC32-UBIp::TurboID-mGFP5* constructs were introduced into WT Col-0 plants using the floral dip method described above. Transgenic seeds were sown on 1/2 MS medium containing 1% agar and 1% sucrose and grown for 10 days under a 16-hour light/8-hour dark cycle in a growth chamber. Seedlings were then harvested, submerged in a 100 μM biotin solution (in water), and incubated at room temperature for 3 hours. After incubation, the biotin solution was removed, and the seedlings were rinsed three times with water to eliminate excess biotin. The seedlings were subsequently dried with paper towel and ground to a fine powder in liquid nitrogen.

For total protein extraction, three biological replicates, each consisting of 10 g of powdered seedlings, were suspended in 20 ml of protein extraction buffer containing 50 mM tris (pH 7.5), 150 mM NaCl, 0.1% SDS, 1% Triton X-100, 0.5% sodium deoxycholate, 1 mM EGTA, 1 mM dithiothreitol (DTT), 1× Protease Inhibitor Cocktail, and 1 mM phenylmethylsulfonyl fluoride. The samples were stirred, vortexed in a cold room for 10 min, and sonicated on ice (Thermo Fisher Scientific, Sonic Dismembrator model 500, 0.5-inch (1.27-cm) probe, 20% amplitude, 10 s on/10 s off for 2 min). The lysate was filtered through Miracloth. The filtrate was centrifuged sequentially at 2000*g* for 10 min and 9000*g* for 10 min at 4°C (Sorvall RC 5C Plus centrifuge, SS-34 rotor). A PD-10 desalting column (GE Healthcare, 17085101) was equilibrated with equilibration buffer [50 mM tris (pH 7.5), 150 mM NaCl, 0.1% SDS, 1% Triton X-100, 0.5% sodium deoxycholate, 1 mM EGTA, and 1 mM DTT] before being used to remove excess free biotin. The eluted protein fraction was incubated with 200 μl of streptavidin-coated magnetic beads (Dynabeads MyOne Streptavidin C1, Invitrogen) overnight at 4°C. The beads were extensively washed, resuspended in 1 ml of extraction buffer, and transferred to a new tube. The samples were digested with trypsin and analyzed by LC-MS/MS on an Orbitrap Eclipse mass spectrometer, as described previously (*67*–*69*) at the Carnegie Mass Spectrometry Facility.

MS/MS spectra were searched against the TAIR10 database, concatenated with decoy protein sequences, using FragPipe v22.0 (*70*). The “LFQ-MBR” workflow built into FragPipe was used, with both precursor and fragment mass tolerances set to 20 parts per million. Methionine oxidation (+15.9949 Da) and protein N-terminal acetylation (+42.0106 Da) were specified as variable modifications, while carbamidomethylation (+57.0215 Da) of cysteine was set as a fixed modification. All other parameters were maintained at their default settings. Statistical analysis of the proteomics data was performed using FragPipe-Analyst (*71*). Proteins were classified as significantly enriched proxiome proteins if they exhibited a *q* value ≤0.05 and a fold enrichment ≥2.

### PUP-IT–based protein association assay in *N. benthamiana*

The constructs of PUP-IT system were adopted from previous reports (*72*, *73*). Briefly, to generate the PUP-IT entry clones, the *CB5LP* coding sequence was PCR-amplified and subcloned into an entry vector of TOPO-UBIp-PafA-hsp at *SalI* and *SpeI* restriction sites to create the expression cassette *UBIp::CB5LP-PafA*. This cassette was subsequently inserted into the destination vector of pMDC32-FLAG-PUP(E) via LR recombination to produce the binary vector coexpressing *35Sp::FLAG-PUP(E)* and *UBIp::CB5LP-PafA*. The *CYP51A2* CDS and the tCNX1 were amplified by PCR, cloned into the *pDONR207* and *pCR8* entry vector, respectively, and sequence-verified to generate the corresponding entry clone. An LR reaction was performed to obtain the expression construct *pEarleyGate101-CYP51A2-YFP-HA* and *pEarleyGate101-tCNX1-YFP-HA*. The resulting binary vectors were introduced into *A. tumefaciens* strain GV3101, and *N. benthamiana* leaves were infiltrated for transient expression. After 3 days of incubation, total proteins were extracted, immunoprecipitated, and analyzed by immunoblotting according to the established procedures (*33*). GFP-Trap Magnetic Agarose beads (GMTA-10, ChromoTek) were used for immunoprecipitation of CYP51A2-YFP-HA. The antibodies used in the immunoblots were anti-HA (HA.11 clone 16B12, Covance), anti-GFP (A-6455, Thermo Fisher Scientific), and anti-FLAG (F3165, Sigma-Aldrich) antibodies. The secondary antibodies were anti–rabbit–horseradish peroxidase (HRP, A9169, Sigma-Aldrich) and anti–mouse-HRP (31430, Thermo Fisher Scientific).

### Protein expression, purification,, and reductase assay

The expression and purification of CBR1, ATR1 and ATR2 were described previously (*34*). For CB5LP and 2muLP, the coding sequences lacking TM domain were PCR-amplified and inserted to pET28a (+) vector via *EcoRI* and *SalI* restriction enzyme sites. The protein expression and purification of CB5LP and 2muLP proteins and the reduction assay were performed following the methods described previously (*34*). Briefly, the verified constructs were introduced into *E. coli* BL21 cells and cultivated overnight at 37°C in 20 ml of Terrific Broth. Subsequently, the precultures were transferred to 600 ml of Terrific Broth and grown at 37°C until reaching an optical density of approximately 1 at 600 nm. The cultures were then shifted to 15°C for 30 min, followed by addition of 0.5 mM IPTG to induce protein expression. The cultures were then harvested after overnight induction and stored at −80°C. Recombinant proteins were purified using Ni^2+^-nitrilotriacetic acid agarose beads (Qiagen) and desalted using Bio-Gel P-6DG gel (Bio-Rad). CB5 protein concentration was determined from the absolute spectrum using an extinction coefficient ε_413_ = 117 mM^−1^ cm^−1^ or from the different spectra of the cytochrome catalyzed by sodium dithionite using an extinction coefficient of ∆ε (reduced-oxidized)_424–409_ = 185 mM^−1^ cm^−1^ (*74*). The purified protein was aliquoted and stored at −80°C for subsequent use. For the redox assay, 100-μl reaction system was used in a 96-well microplate, containing 20 mM tris-HCl buffer (pH 7.5), and varied amount of purified CB5 and CBR or ATR2 proteins. The oxidized and reduced status of CB5 proteins were monitored before and after the addition of NADH or NADPH, respectively.

### Yeast microsome preparation and CYP51A2 enzyme activity assay

All constructs used for yeast expression were generated using *pYeDP60* vector (*75*), which was digested with *BamHI* and *EcoRI* restriction enzymes. The coding sequences of target genes were amplified by PCR with the specific fusion primers (table S1) and inserted to *pYeDP60* vector via Gibson assembly method (*76*). Constructs were transformed into a yeast strain deficient in its endogenous *CB5* (*cyb5*, MAT-a, Transomic), and positive colonies were selected on auxotrophic yeast medium lacking uracil. Yeast induction and microsome preparation were performed as described previously (*34*). The substrate obtusifoliol was dissolved in methyl-β-cyclodextrin (Sigma-Aldrich, C4555): ethyl alcohol (5:100, v/v) at concentration of 0.5 μg/μl. Microsome enzyme activity assays were performed in 2-ml centrifuge tubes. Each 1-ml reaction mixture contained 100 mM phosphate-buffered saline buffer (pH7.5), with 20% glycerol, 400 μg of microsomal protein, 2.5 μg of obtusifoliol, 0.2 mM NADH, or 0.2 mM NADPH. Control reactions were performed in the absence of NADH or NADPH. After 3 hours of incubation at 28°C with continuous rotation, the reaction products were extracted using ethyl acetate containing 5-α-cholestene as internal standard. Extracts were vacuum-dried, resuspended in 50 μl of pyridine and 50 μl *N*-methyl-*N*-(trimethylsilyl) trifluoroacetamide, and incubated at 70°C for 1 hour. One microliter of the product was injected into GC-MS, following the sterol analysis method described earlier.

### Phylogenetic analysis

The plant CB5 and CB5LP homologs sequences were retrieved using BLASTP search with AtCB5LP (At1g60660) or AtCB5D (At5g48810) as the initial queries. Searches were conducted against Phytozome 13 (*C. reinhardtii*, *V. carteri*, *M. polymorpha*, *P. patens*, *S. moellendorffii*, *C. richardii*, *A. trichopoda*, *O. sativa*, and *P. trichocarpa*), FernBase (*S. cucullata*), Genbank (*P. purpureum*, *G. domingensis*, *R. marinus*, *C. caldarium*, *G. chorda*, and *C. merolae*), ONEKP (*C. vulgaris*, *P.* radiata, and *G. biloba*), and Solanaceae Genomics Network (*P. axillaris*). Then, the resulting sequences were reciprocally blasted (via BLASTP) against the *A. thaliana* protein database and hits were retained if the best matches were *AtCB5A*, *AtCB5B*, *AtCB5C*, *AtCB5D*, *AtCB5E*, or *AtCB5LP*.

Homologs of CB5 and CB5LP from Metazoa, Fungi, and Bacteria were obtained using BLASTP search against Clustered nr (nr_clustered) database (https://blast.ncbi.nlm.nih.gov/) with *AtCB5LP* as the query. The resulting sequences were analyzed using TMHMM-2.0 (*41*) to predict the TM helices and localization. The cytochrome *b_5_* domain was extracted using the HMMER algorithm (https://hmmer.org/). Hits were retained for further analysis if they met the following criteria: peptide lengths between 100 and 200 amino acids, start codon as methionine, cyt-*b_5_* domain length of at least 70 amino acids, and a maximum of one TM helix.

For phylogenetic analysis, the full-length peptide sequences or cyt-*b_5_* domain sequences were aligned using MUSCLE algorithm integrated within the MEGA X program (*77*). Phylogenetic trees were inferred using the maximum likelihood method implemented in IQ-TREE-2.2.2.7 program (*78*, *79*). The following command line was used for tree reconstruction: “bin\iqtree2 -s alignment.FAS -alrt 1000 -B 1000” (data S13). The best model was selected using the integrated ModelFinder algorithm. To assess branch support, 1000 ultrafast bootstrap replicates and 1000 Shimodaira-Hasegawa approximate likelihood ratio tests were performed. The tree visualization and annotation were performed using TVBOT (*80*).

### Statistical analysis

Statistical analysis for each required experiment was performed using either Student’s *t* test in Microsoft Excel (two-tailed distribution and two-sample unequal variance) or analysis of variance (ANOVA) test in GraphPad Prism version 4 (*P* < 0.05, one-way ANOVA and Tukey’s test). Details of statistical analyses, including sample sizes and biological replicates, are provided in the figure legends.

### Accession numbers

DNA and derived protein sequence data from this article are available in the TAIR database (https://arabidopsis.org/), Genebank (https://ncbi.nlm.nih.gov/genbank/), Phytozome 13 (https://phytozome-next.jgi.doe.gov/), Solanaceae Genomics Network (https://solgenomics.net/) and FernBase (https://fernbase.org/). The *Arabidopsis* genes are under the following TAIR accession numbers: *AtCB5A* (At1g26340), *AtCB5B* (AT2G32720), *AtCB5C* (At2g46650), *AtCB5D* (At5g48810), *AtCB5E* (At5g53560), *AtCB5LP* (At1g60660), *CBR1* (At5g17770), *ATR1* (At4g24520); *ATR2* (At4g30210), and *CYP51A2* (At1g11680).
