# Supplementary material for: A plant-specific cytochrome b5–like protein is essential for phytosterol biosynthesis
Source: Sci Adv. 2025 Sep 10;11(37):eady1719. doi: 10.1126/sciadv.ady1719 (PMC13155568; doi:10.1126/sciadv.ady1719)
Supplement: Supplementary file 1 — Figs. S1 to S19 Table S1 Legend for data S1 to S13 [file sciadv.ady1719_sm.pdf]

Science Advances
AAAS

Supplementary Materials for

A plant-specific cytochrome b_5–like protein is essential for phytosterol biosynthesis

Xianhai Zhao *et al.*

Corresponding author: Chang-Jun Liu, cliu@bnl.gov

Sci. Adv. **11**, eady1719 (2025)
DOI: 10.1126/sciadv.ady1719

The PDF file includes:

Figs. S1 to S19
Table S1
Legends for data S1 to S13

Other Supplementary Material for this manuscript includes the following:

Data S1 to S13

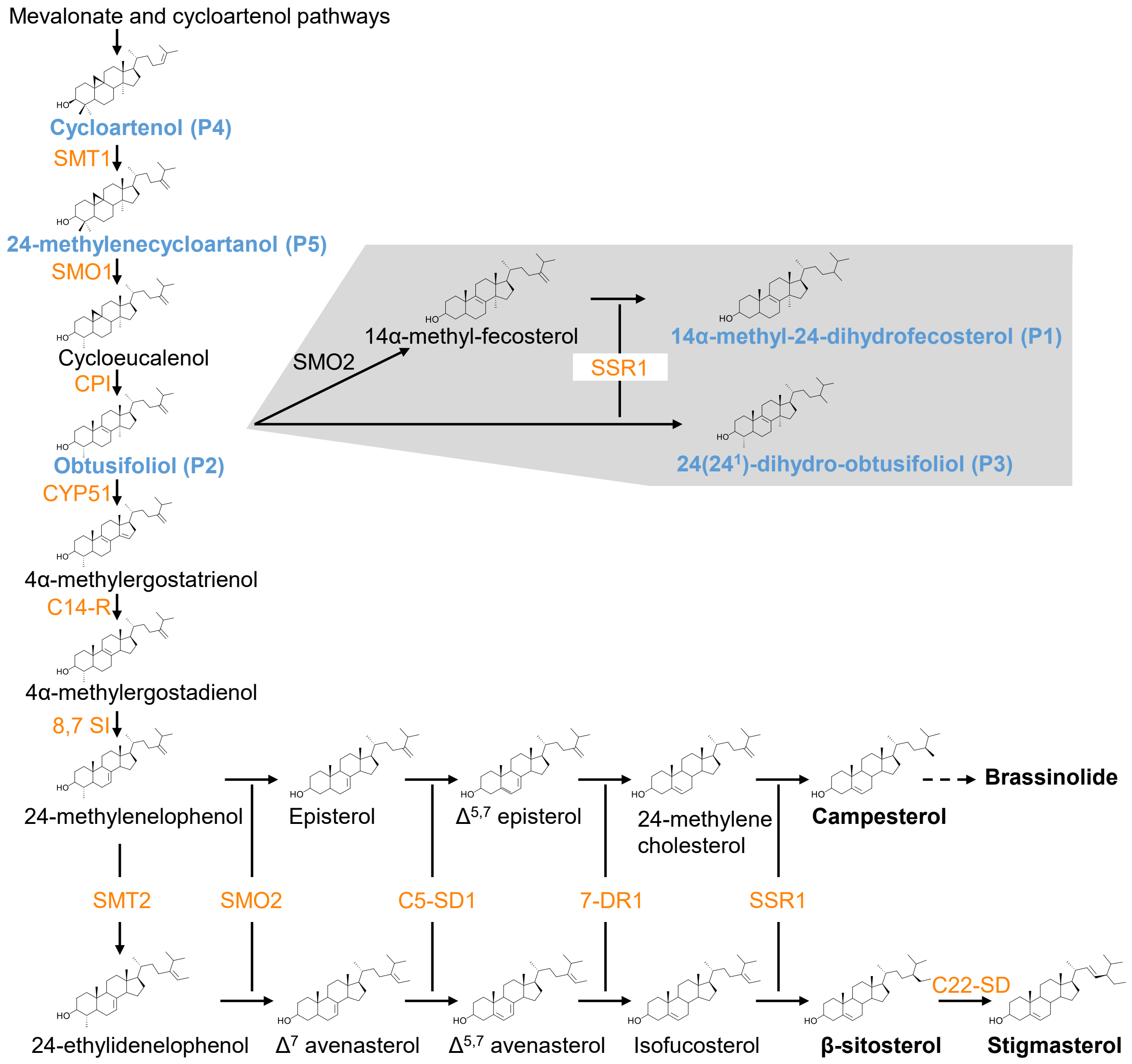

Fig. S1. The phytosterol biosynthetic pathway in *Arabidopsis*. The deduced pathway specific to the *CB5LP* mutant is shaded. Five accumulated metabolites (P1-P5) detected in the *CB5LP* mutant are highlighted in blue. SMT, sterol C-24 methyltransferase; SMO, C-4 sterol methyl oxidase; CPI, cyclopropylsterol isomerase; CYP51, sterol C-14 demethylase; C14-R, sterol C-14 reductase; 8,7 SI, sterol 8,7 isomerase; C5-SD1, sterol C-5(6) desaturase 1 (DWARF7); 7-DR2, 7-dehydrocholesterol reductase 2; 7-DR1, 7-dehydrocholesterol reductase 1 (DWARF5); SSR1, sterol side chain reductase 1; C22-SD, C22-sterol desaturase.

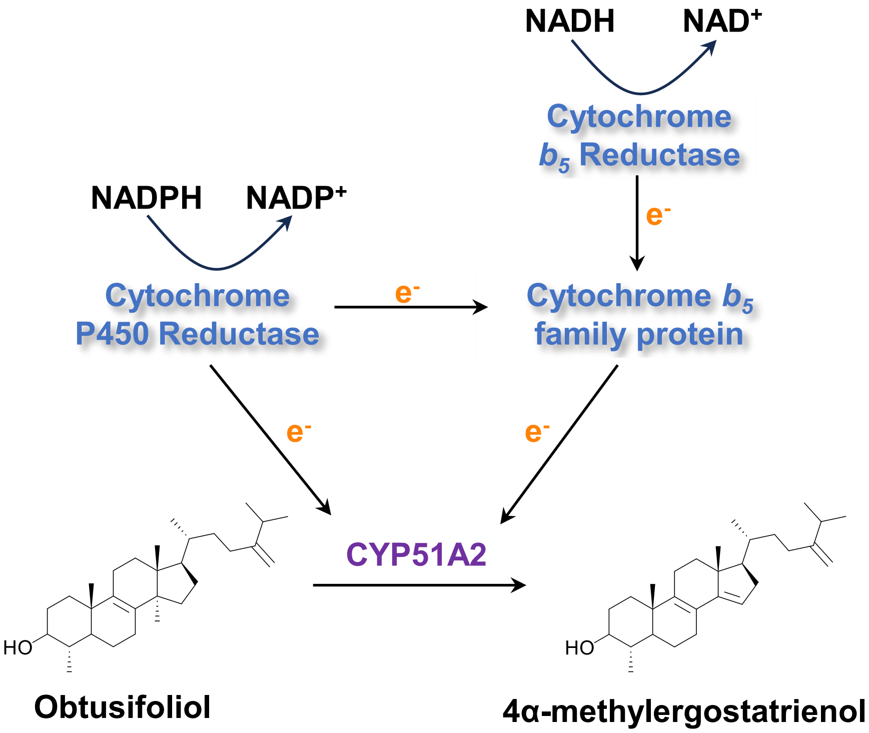

Fig. S2. Electron transfer chains in the endoplasmic reticulum that supply reducing equivalents to CYP51A2.

A

	AtCB5A	AtCB5B	AtCB5C	AtCB5D	AtCB5E	AtCB5LP
AtCB5A	100					
AtCB5B	43.41	100				
AtCB5C	47.2	43.85	100			
AtCB5D	39.53	67.91	45.45	100		
AtCB5E	47.29	61.94	48.46	59.7	100	
AtCB5LP	46.25	41.25	36.36	38.75	41.25	100

B

	AtCB5A 9-80	AtCB5B 9-80	AtCB5C 6-77	AtCB5D 9-80	AtCB5E 9-80	AtCB5LP 50-120
AtCB5A 9-80	100					
AtCB5B 9-80	59.72	100				
AtCB5C 6-77	63.89	51.39	100			
AtCB5D 9-80	52.78	75	52.78	100		
AtCB5E 9-80	65.28	68.06	59.72	68.06	100	
AtCB5LP 50-120	49.3	45.07	39.44	42.25	45.07	100

C

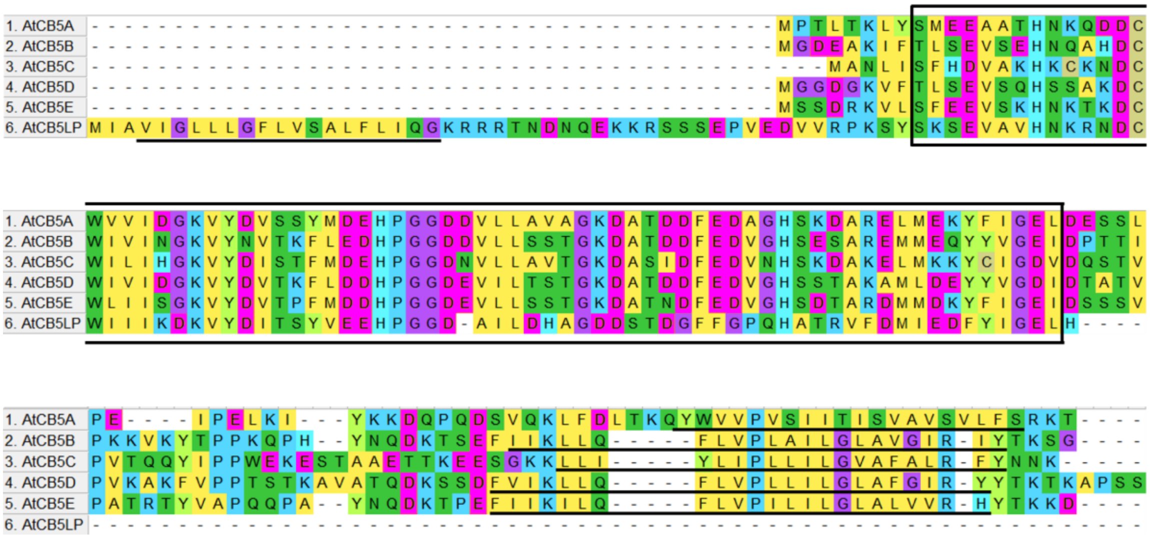

Fig. S3. Percent identity matrix and multiple sequence alignment of *Arabidopsis* CB5 proteins. (**A** and **B**) Percent identity matrices of the full-length sequences (A) and their cytochrome b_5 (cyt-b_5) domain sequences (B) of AtCB5 and AtCB5LP proteins. (**C**) Multiple sequence alignment of the five canonical CB5 proteins (AtCB5A to AtCB5E) and one CB5 like protein (AtCB5LP) in *Arabidopsis*. The transmembrane domains are underlined, while the cyt-b_5 domains are boxed.

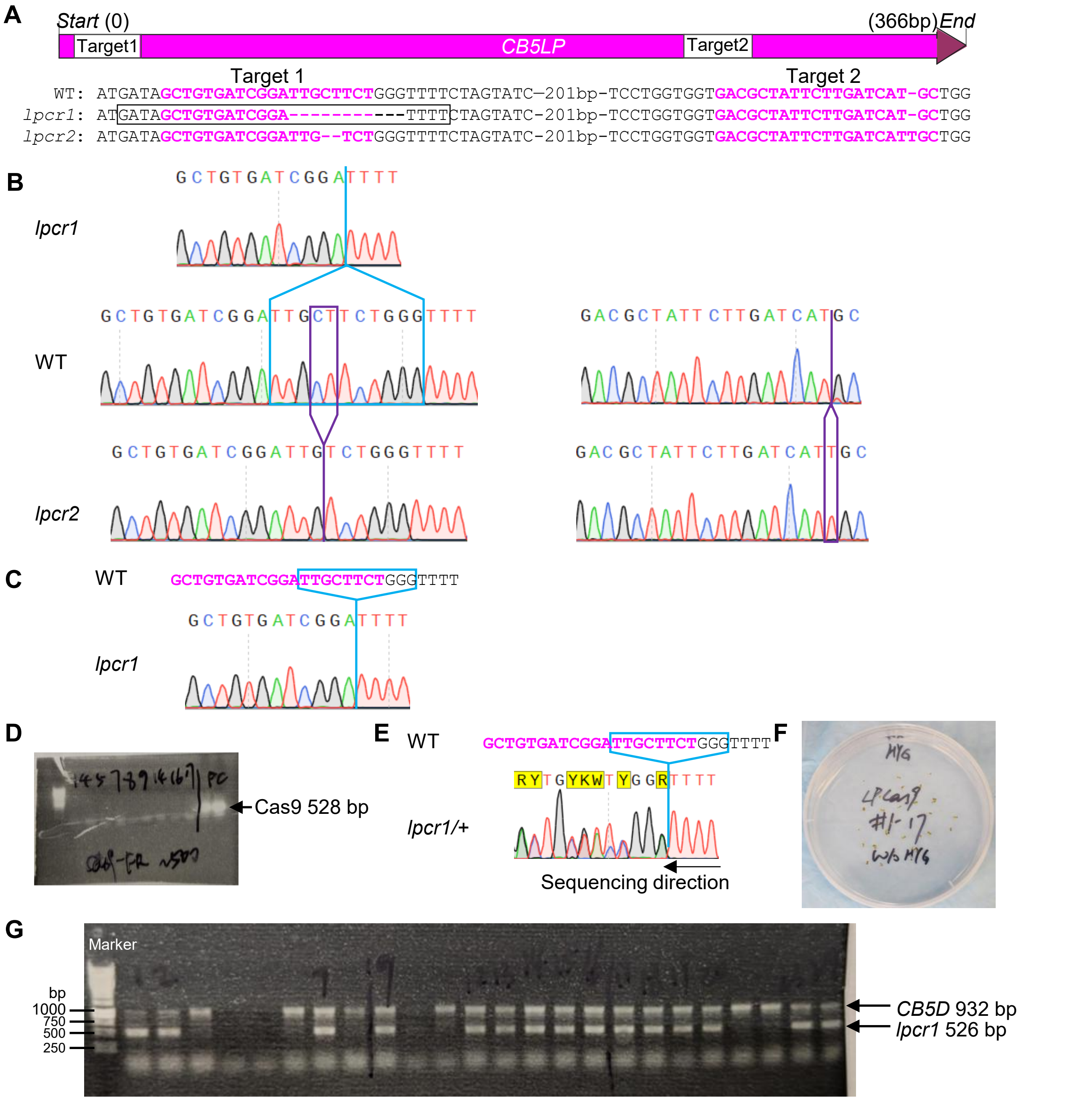

Fig. S4. CRISPR/Cas9 gene editing to generate *lpcr* mutants.
(**A**) Schematic representation of *CB5LP* gene showing the two CRISPR target sites and their corresponding mutations in the knockout *lpcr* mutants, *lpcr1* and *lpcr2*. The boxed oligos represent primers designed for PCR genotyping of *lpcr1* allele. (**B**) Sanger sequencing chromatograms confirming the mutations in the obtained homozygous *lpcr* mutants. (**C**) Sanger sequencing chromatogram showing the *cb5lp* mutation in the plants germinated from twisted *lpcr1* seeds. (**D**) to (**F**) Isolation of T-DNA free *lpcr1/+* mutants. F2 seeds derived from backcrossed plants of *lpcr1/+* with WT were germinated on non-selective medium and transferred to soil. PCR genotyping using Cas9-specific primers was performed to identify plants lacking T-DNA insertion (D). The *lpcr1* locus was further sequenced to confirm heterozygosity (*lpcr1/+*) in T-DNA-free individuals (E). To validate the absence of T-DNA, F3 seeds were germinated on 1/2 MS medium containing hygromycin; T-DNA-free lines were identified by their hygromycin sensitivity (F). (**G**) PCR genotyping of *lpcr1* allele. A forward primer exactly matching the *lpcr1* allele sequence at CRISPR target 1 region of *CB5LP* (boxed in panel A), combined with a downstream reverse primer, was designed to amplify the *lpcr1* allele. The CB5D gene was amplified in the same PCR reaction to verify the quality of the DNA template. Samples producing both amplicons were confirmed as true *lpcr1/+* heterozygotes.

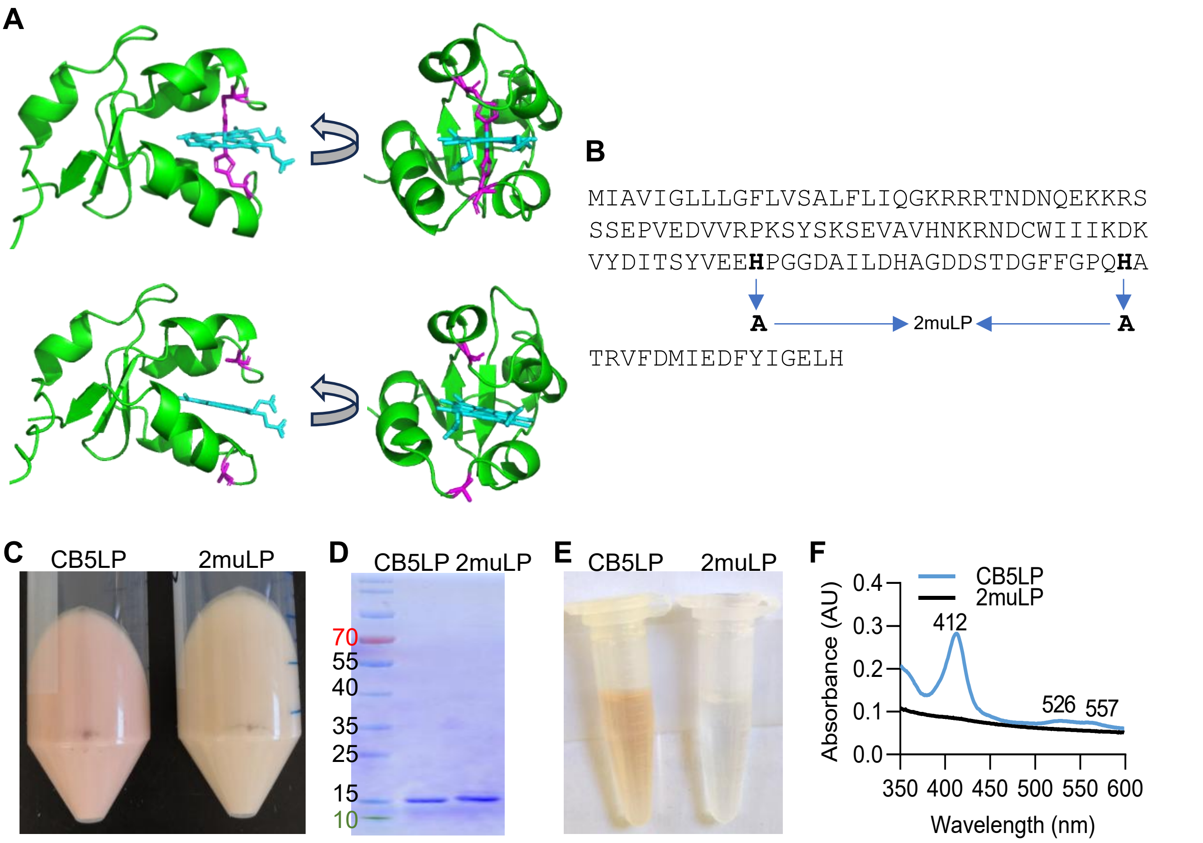

Fig. S5. Preparation of the recombinant CB5LP and 2muLP proteins.
(**A**) AlphaFold3-predicted structures of CB5LP (top) and 2muLP (bottom). The bound heme and two interacting histidine (His) residues are represented as sticks in cyan and magenta, respectively. (**B**) Diagram of the substitutions of His-47 and His-70 in CB5LP to alanine residues in 2muLP. (**C**) Photograph of bacterial cultures after induction for expression of CB5LP and 2muLP proteins. (**D**) SDS-PAGE gel image showing the purified CB5LP and 2muLP proteins. (**E**) Visual coloration of the purified CB5LP and 2muLP proteins. CB5LP exhibits a reddish color characteristic of hemoproteins. (**F**) Absorption spectra of purified recombinant CB5LP (9 μM, blue line) and 2muLP (9 μM, black line).

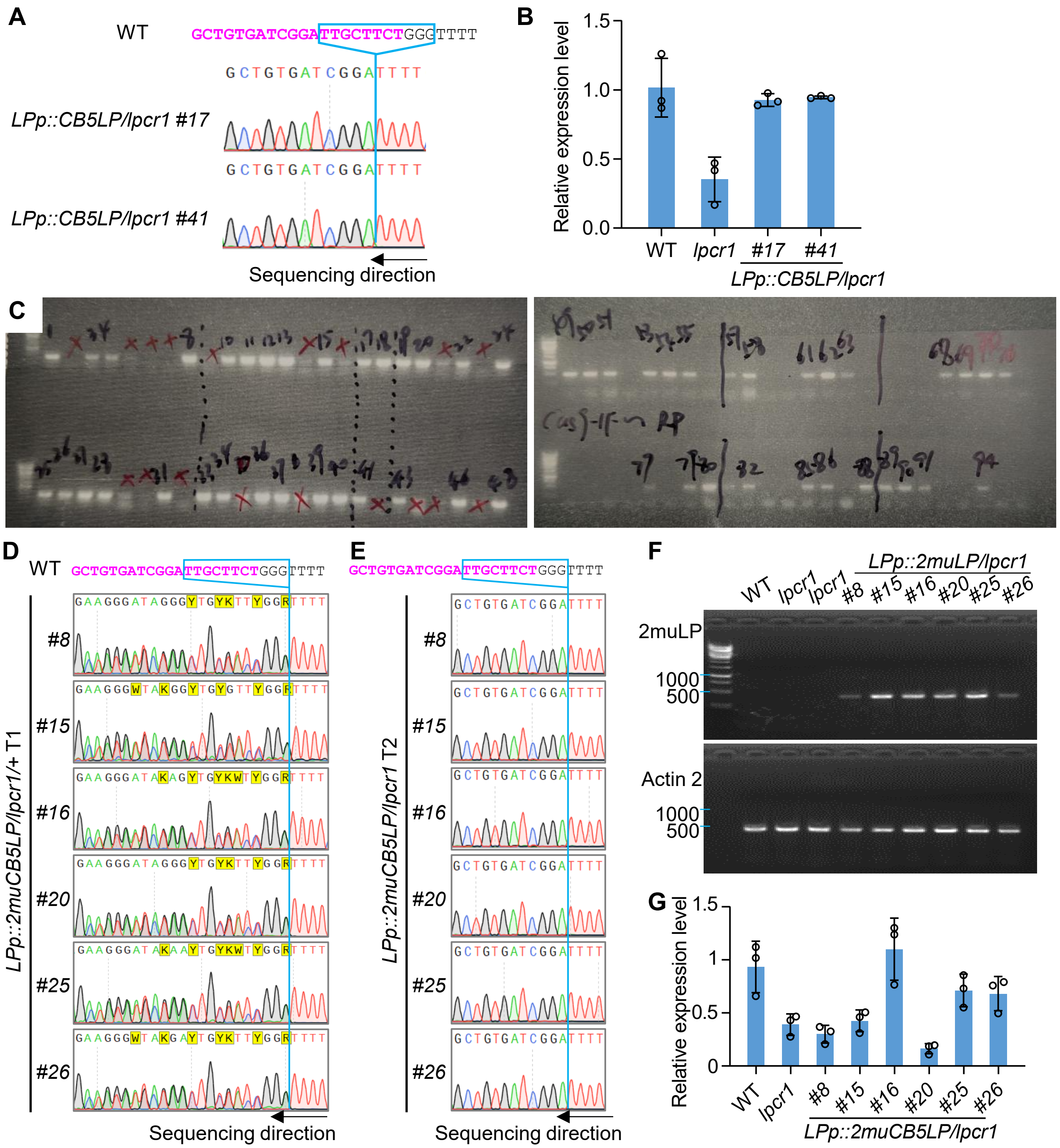

Fig. S6. Confirmation of the *LPp::CB5LP/lpcr1* and *LPp::2muLP/lpcr1* transgenic lines in Fig. 4. (**A**) Sanger sequencing confirming the *lpcr1 h*omozygous background of T1 generation *LPp::CB5LP/lpcr1* transgenic lines. Representative lines *#17* and *#41* are shown. (**B**) RT-qPCR analysis of *CB5LP* transgene expression in T2 seedlings of lines *#17* and *#41*. RNAs were extracted from 1-week-old seedlings. Expression levels were normalized to that of *PP2A* gene, and the expression level of WT was set as 1. Data are presented as means ± SD from three biological replicates. (**C** to **G**) Genotyping and transgene expression analysis of *LPp::2muLP/lpcr1* transgenic lines. (C) PCR genotyping confirmed the presence of the *lpcr1* allele in T1 generation. (D) Sanger sequencing verified the heterozygous *lpcr1/+* background of the normal-sized representative transgenic lines in T1 generation. (E) In T2 generation, Sanger sequencing validated the *lpcr1-type* seedlings shown in Fig. 4G from six representative *LPp::2muLP* transgenic lines in the *lpcr1* homozygous background. (F) RT-PCR analysis confirmed the expression of the *2muLP* transgene in these lines using primers specific to the exogenous 2muLP sequence. (G) RT-qPCR analysis measured the relative expression levels of CB5LP in these lines. RNAs were extracted from 1-week-old seedlings. The expression levels were normalized to that of *PP2A* gene, and the expression level of WT was set as 1. Data are presented as means ± SD from three biological replicates.

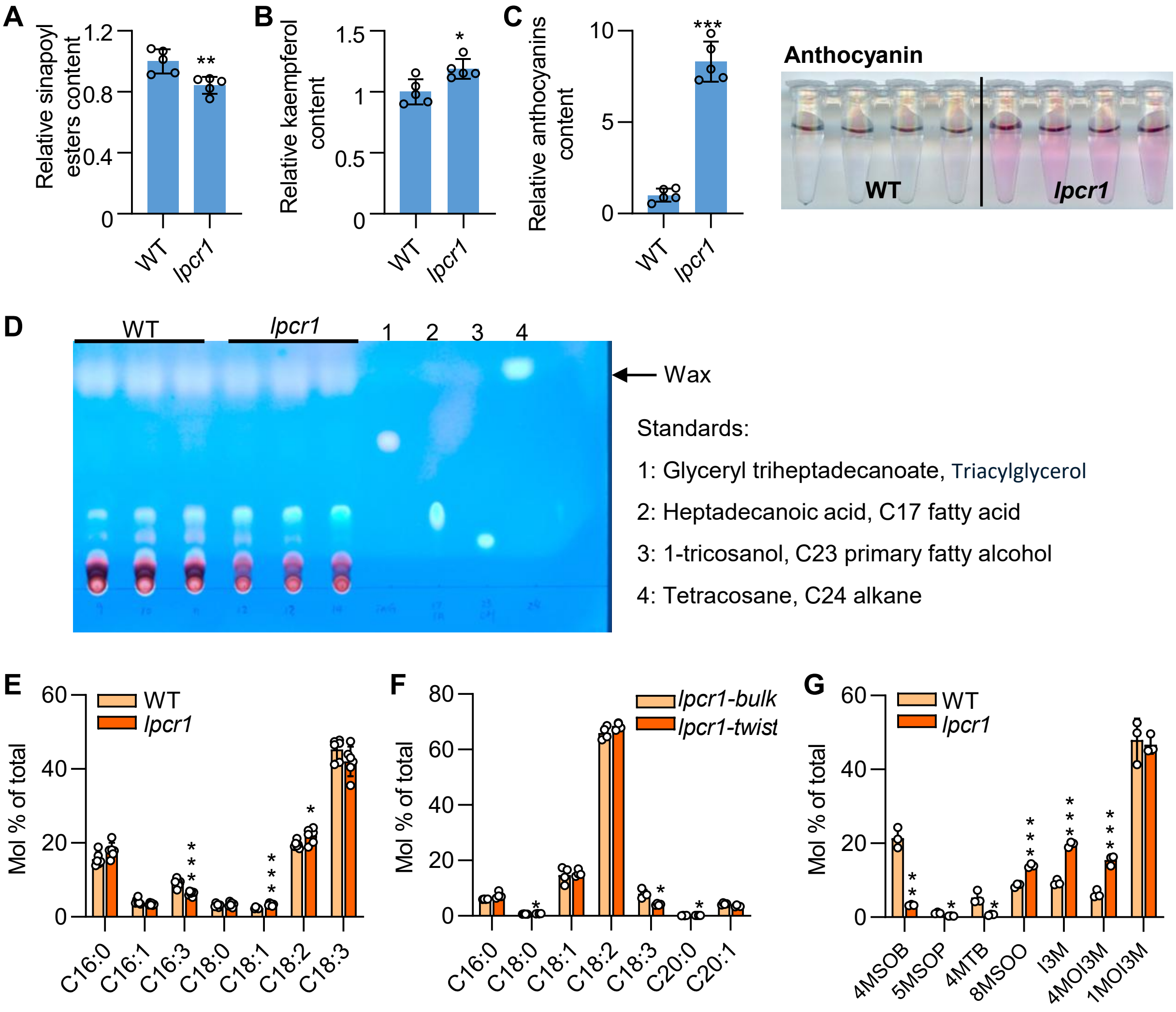

Fig. S7. Targeted metabolite profiling in the WT and *cb5lp* mutant.
(**A** to **C**) Relative levels of sinapoyl esters (A), kaempferol (B), and anthocyanin (C) in 10-day-old seedlings of WT and *lpcr1* mutant. The levels of WT were normalized to 1. (**D**) Thin-layer chromatography of lipid extracts from 14-day-old seedlings of WT and *lpcr1* mutant. (**E**) Fatty acid composition in 2-week-old seedlings of WT and *lpcr1* mutant. (**F**) Fatty acid composition in mature seeds of *lpcr1/+* progeny, analyzed separately for bulk seeds and twisted seeds. (**G**) Glucosinolate composition in 14-day-old seedlings of WT and *lpcr1* mutant. 4MSOB, 4-methylsulfinylbutyl; 5MSOP, 5-methylsulfinylpropyl; 4MTB, 4-methylthiobutyl; 8MSOO, 8-methylsulfinyloctyl; I3M, indol-3-ylmethyl; 4MOI3M, 4-methoxyindole-3-ylmethyl; 1MOI3M, 1-methoxyindole-3-ylmethyl. Data are presented as means ± SD from five (A to C), six (E), four (F), or three (G) biological replicates. Asterisks denote significant differences with *$P < 0.05$, **$P < 0.01$, and ***$P < 0.001$ (two-tailed Student's t tests).

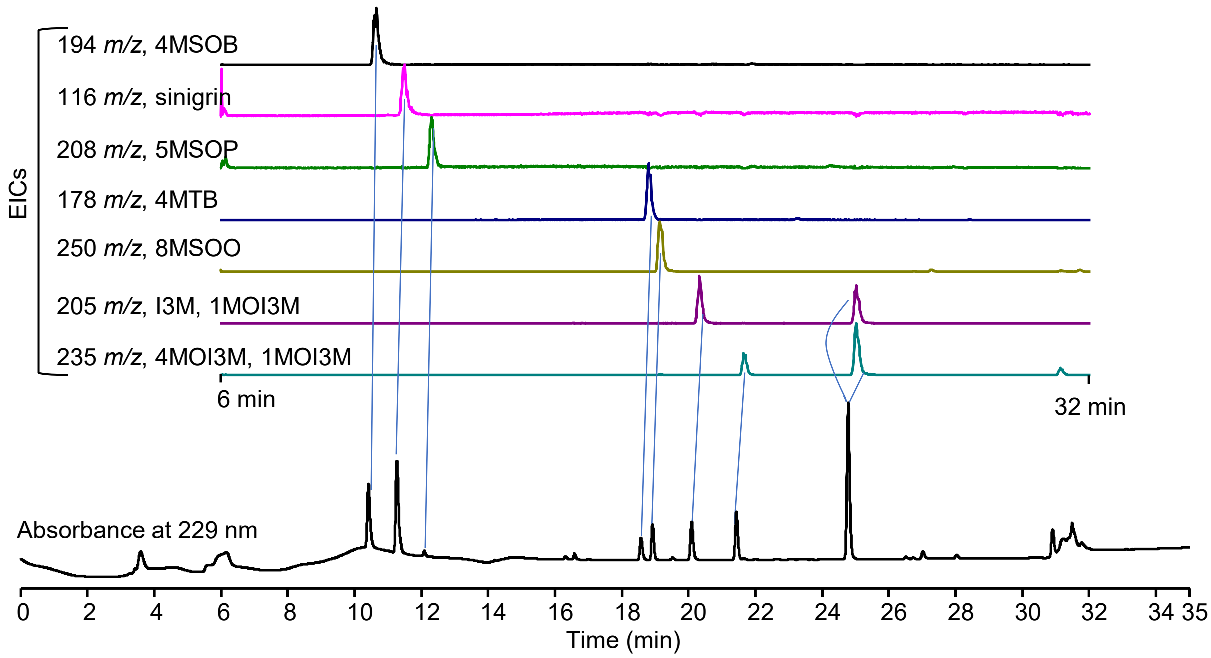

Fig. S8. UHPLC-MS analysis of glucosinolates from *Arabidopsis* seedlings.
Chromatograms shows UHPLC-UV absorptive profile of desulfoglucosinolates extracted from 2-week-old *Arabidopsis* seedlings, monitored at 229 nm, along with the extracted ion chromatograms of the detected desulfoglucosinolate compounds. The identified glucosinolates included 4MSOB, 4-methylsulfinylbutyl; 5MSOP, 5-methylsulfinylpropyl; 4MTB, 4-methylthiobutyl; 8MSOO, 8-methylsulfinyloctyl; I3M, indol-3-ylmethyl; 1MOI3M, 1-methoxyindol-3-ylmethyl; and 4MOI3M, 4-methoxyindol-3-ylmethyl. Sinigrin was used as an internal standard.

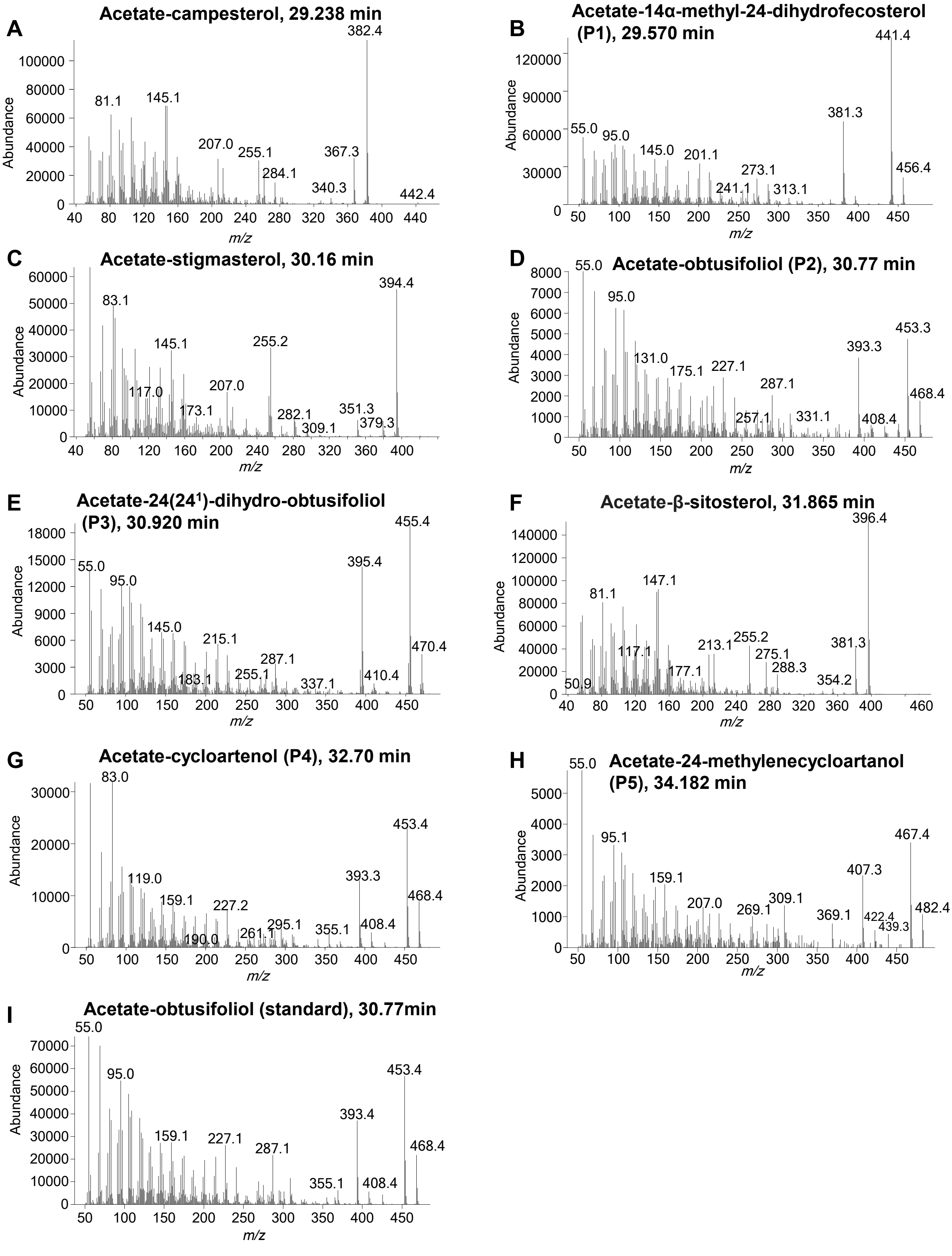

Fig. S9. Mass spectra of identified sterols in *Arabidopsis*.
(**A** to **H**) Mass spectra of eight sterols identified in WT and *lpcr1* plants. (**I**) Mass spectrum of the Obtusifoliol standard. Sterol compounds were derivatized as acetate esters prior to GC-MS analysis.

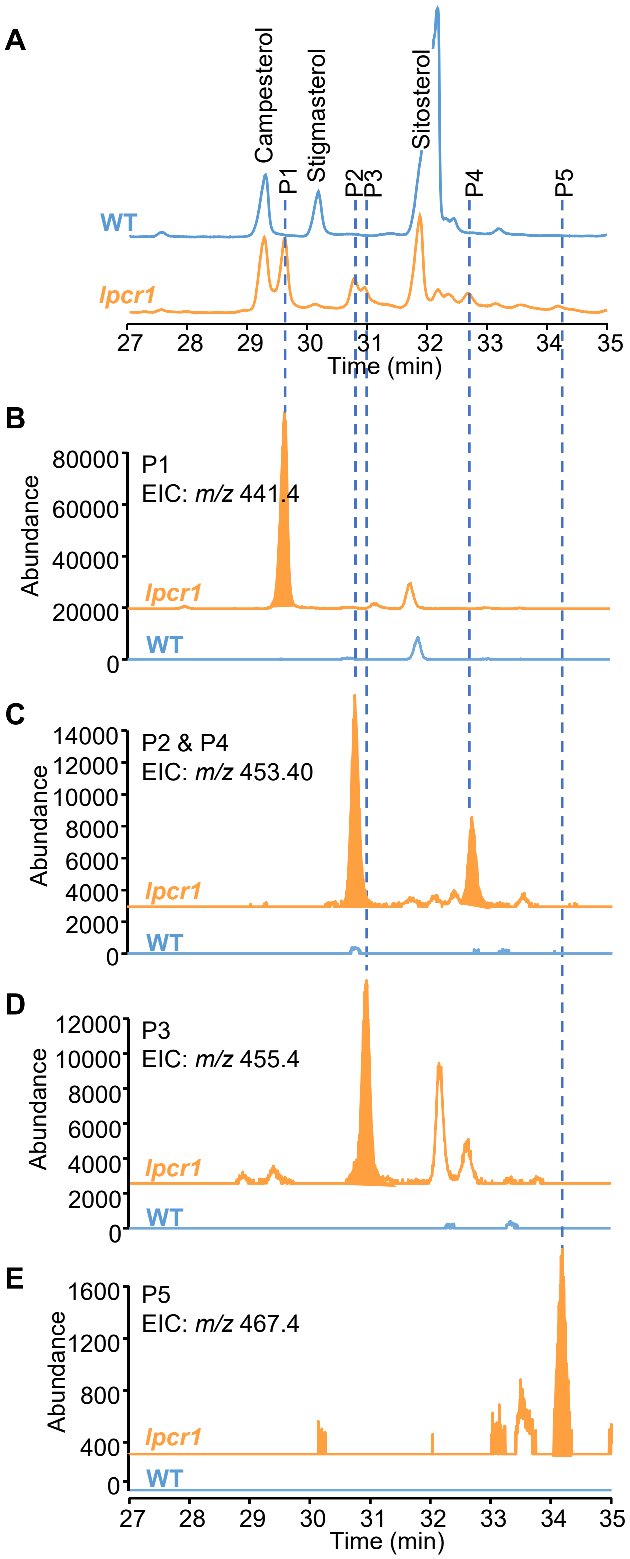

Fig. S10. GC-MS profile of sterols in the *lpcr1* mutant.
(**A**) Total ion chromatograms of sterol profile in the *lpcr1* seedlings as displayed in Fig.5A. (**B** to **E**) Extracted ion chromatograms (EICs) showing P1 (B), P2 and P4 (C), P3 (D), and P5 (E) in the *lpcr1* and WT. P1, 14α-methyl-24-dihydrofecosterol; P2, Obtusifoliol; P3, 24(24^{1})-dihydro-obtusifoliol; P4, Cycloartenol; and P5, 24-methylenecycloartanol.

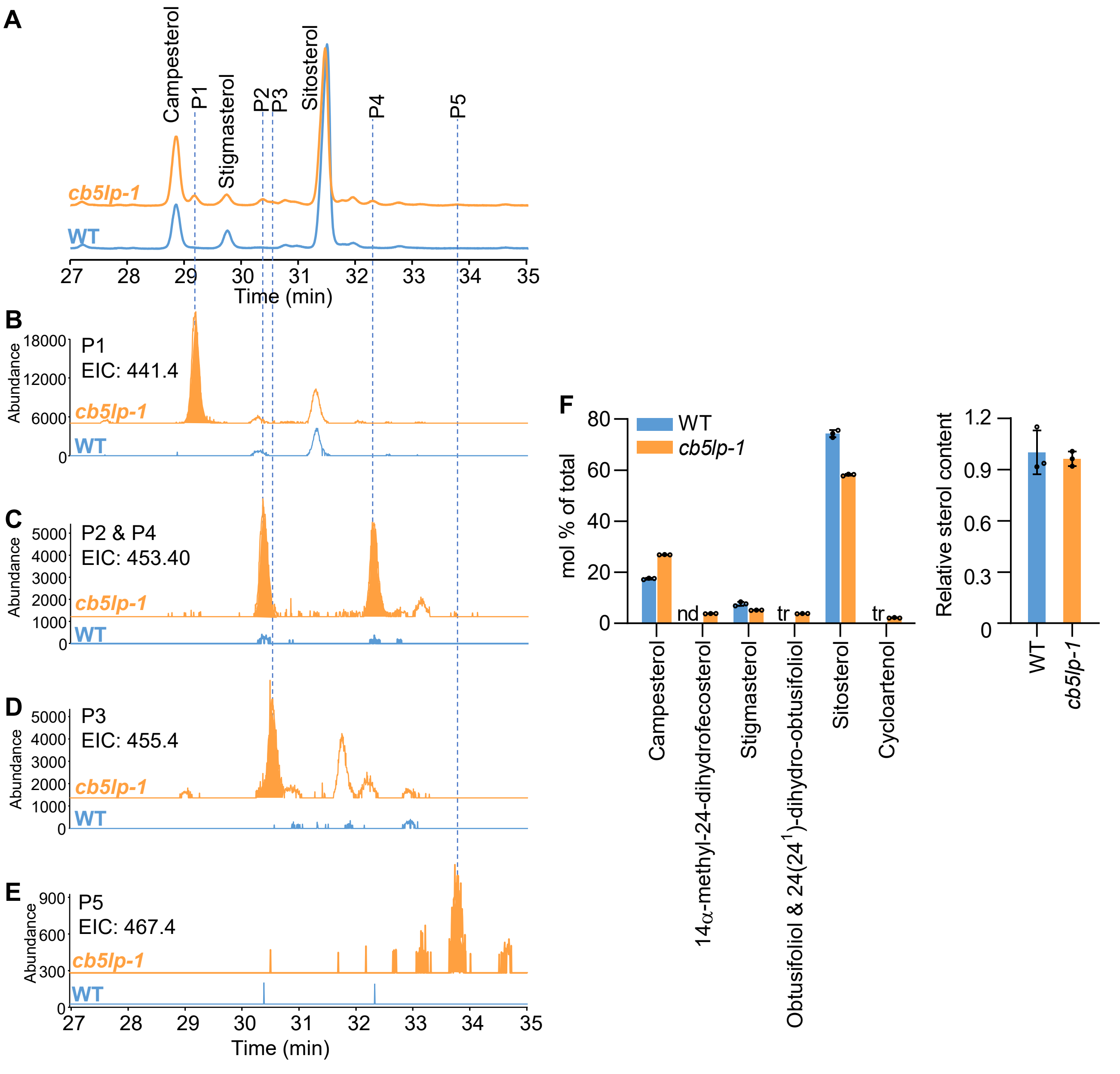

Fig. S11. Sterol composition in the *cb5lp-1* knockdown mutant.
(**A**) GC-MS total ion chromatograms of sterol profiles from 10-day-old *Arabidopsis* seedlings of *cb5lp-1* mutant and WT. P1, 14α-methyl-24-dihydrofecosterol; P2, Obtusifoliol; P3, 24(24^1)-dihydro-obtusifoliol; P4, Cycloartenol; and P5, 24-methylenecycloartanol. (**B** to **E**) Extracted ion chromatograms (EICs) of sterol compounds: P1 (B), P2 and P4 (C), P3 (D), and P5 (E), comparing *cb5lp-1* and WT seedlings. (**F**) Quantification of sterol composition and total sterol content in 10-day-old seedlings of the WT and *cb5lp-1* lines. nd, not detected; tr, trace amount. Data are presented as means ± SD from three biological replicates.

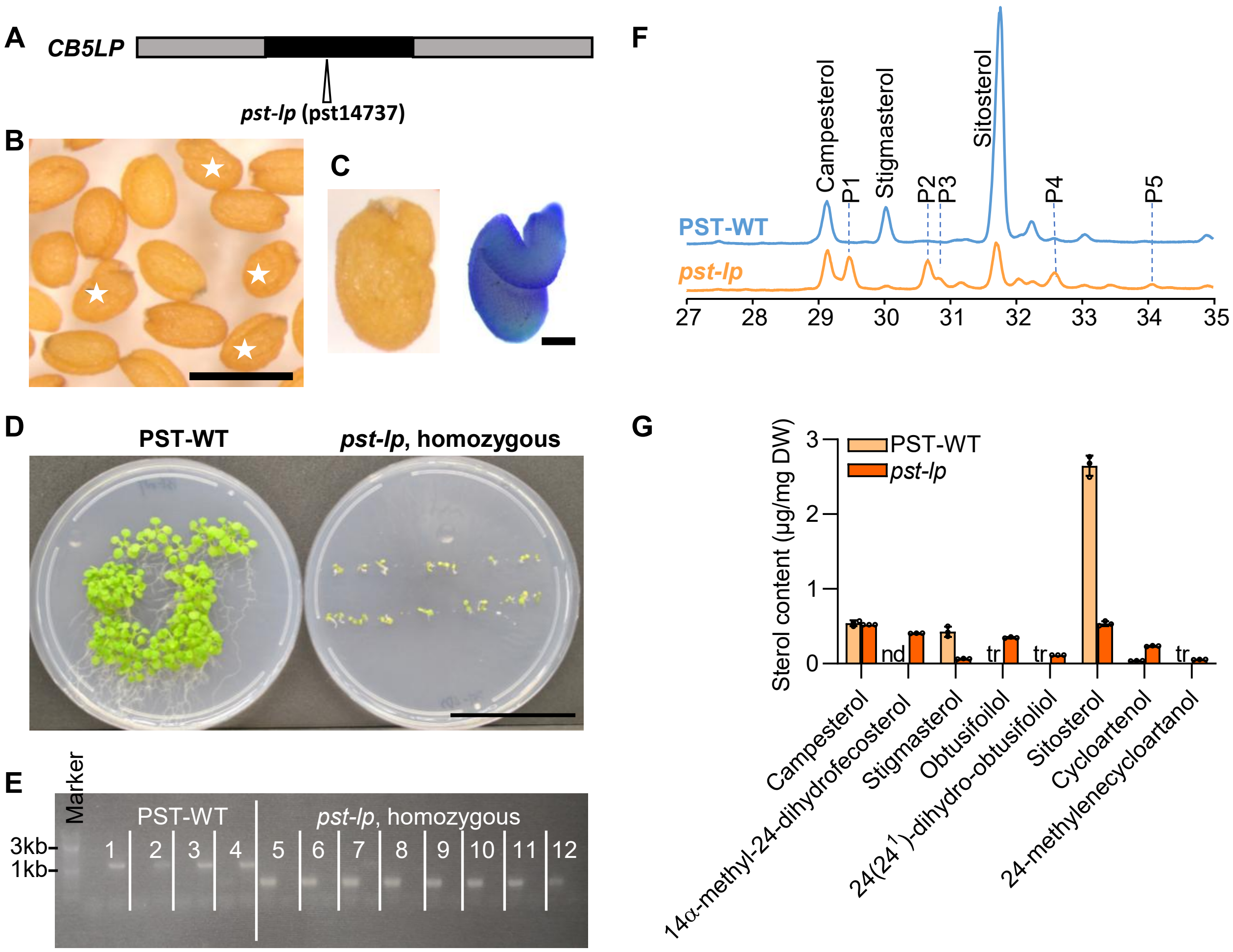

Fig. S12. Characterization of *CB5LP* transposon-insertion mutant line.
(**A**) Diagram of transposon insertion mutant of *CB5LP* showing the insertion site (triangle). The left and right gray boxes indicate 5' and 3' untranslated regions, and black box indicates the single exon of CB5LP. The Nossen-0 background line (CS1394) was obtained from ABRC and named PST-WT, while the transposon-insertion line (*pst14737*) was obtained from RIKEN and named *pst-lp*. (**B**) Morphology of dry mature seeds of *pst-lp/+* progeny. The seeds with twisted phenotype are indicated with white stars. (**C**) Morphology of twisted *pst-lp/+* progeny seeds with (left) and without (right) seed coats. After seed coat removal, embryos were stained with toluidine blue for observation. (**D**) Morphology of 2-week-old PST-WT (left) and *pst-lp* mutant plants germinated from twisted seeds (right). (**E**) PCR genotyping of PST-WT and *pst-lp* homozygous seedlings in panel (D). Each pair of adjacent lanes on the agarose gel represents LB + RP and LP + RP PCR products from same DNA sample. (**F**) GC-MS total ion chromatograms showing sterol profiles of 10-day-old seedlings of *pst-lp* mutant and the PST-WT. P1, 14α-methyl-24-dihydrofecosterol; P2, Obtusifoliol; P3, 24(24^1)-dihydro-obtusifoliol; P4, Cycloartenol; and P5, 24-methylenecycloartanol. (**G**) Quantification of sterol composition in 10-day-old seedlings of PST-WT and *pst-lp.* nd, not detected; tr, trace amount. Data are presented as means ± SD from three biological replicates. Scale bars, 0.5 mm (B), 0.1 mm (C) and 5 cm (D).

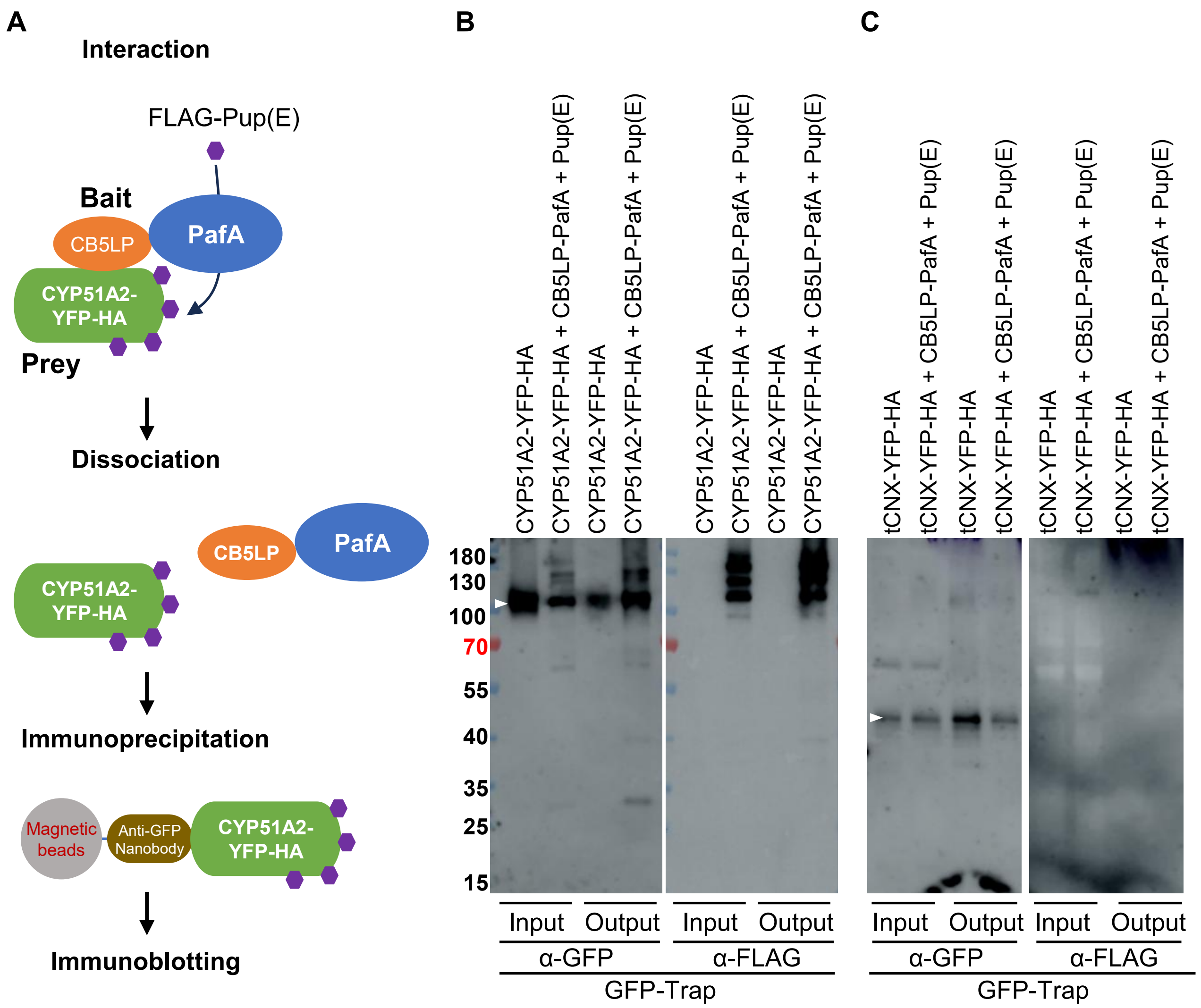

Fig. S13. Validation of protein-protein interaction between CB5LP and CYP51A2.
(**A**) Schematic representation of the pupylation-based interaction tagging proximity-labeling system. The bacterial Pup ligase PafA is fused to CB5LP (bait), allowing covalent attachment of Flag-Pup(E) to lysine residues of interacting proteins such as CYP51A2 (prey). (**B** and **C**) Co-expression of CB5LP-PafA, FLAG-Pup(E), and CYP51A2-YFP-HA (B) or tCNX1-YFP-HA endoplasmic reticulum marker (C) in *N. benthamiana* resulted in FLAG labeling of CYP51A2-YFP-HA, but not tCNX1-YFP-HA. The triangles indicate the expressed fusion proteins. Immunoprecipitation was performed using GFP-trap beads, followed by immunoblotting with α-GFP and α-FLAG antibodies.

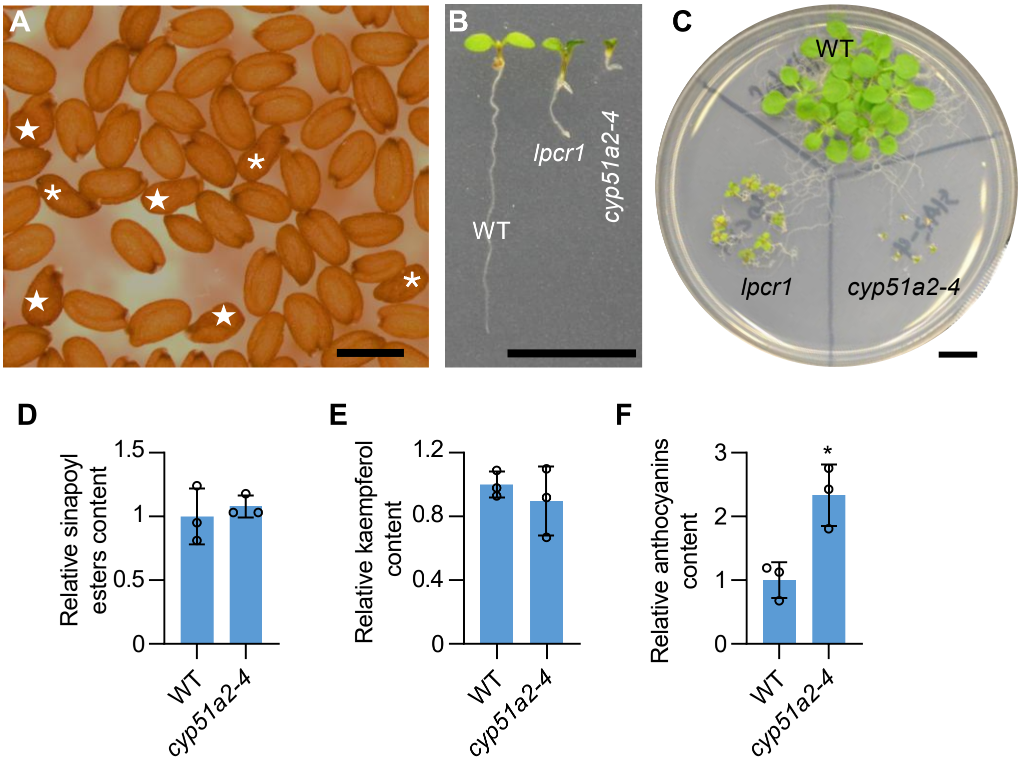

Fig. S14. Characterization of the *cyp51a2-4* mutant.
(**A**) Morphology of dry mature seeds of *cyp51a2-4/+* progeny. Twisted and shriveled seeds are indicated by white stars and asterisks, respectively. (**B and C**) Morphology of 5-day-old (B) and 17-day-old seedlings of WT, *lpcr1* and *cyp51a2-4*. Scale bars, 0.5 mm (A) and 1 cm (B and C). (**D** to **F**) Relative contents of sinapoyl esters (D), kaempferol (E), and anthocyanins (F) in 10-day-old seedlings of WT and *cyp51a2-4* mutant. WT levels were normalized to 1. Data are presented as means ± SD from three biological replicates. Asterisk denotes significant differences with $*P < 0.05$ (two-tailed Student's *t* tests).

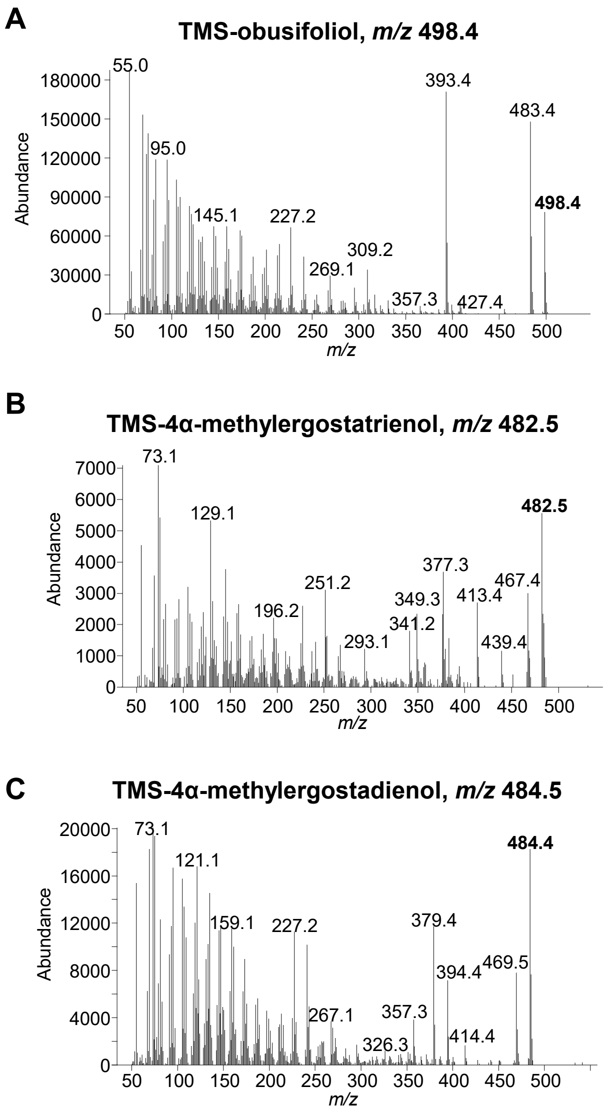

Fig. S15. Mass spectra of the detected products in CYP51A2-catalyzed demethylation reaction of obtusifoliol.

Mass spectrum of substrate Obtusifoliol (**A**) and the two detected products, 4α-methylergostatrienol (**B**) and 4α-methylergostadienol (**C**). The sterol compounds were derivatized as the 3-trimethylsilyl (TMS) ethers before analysis via GC-MS.

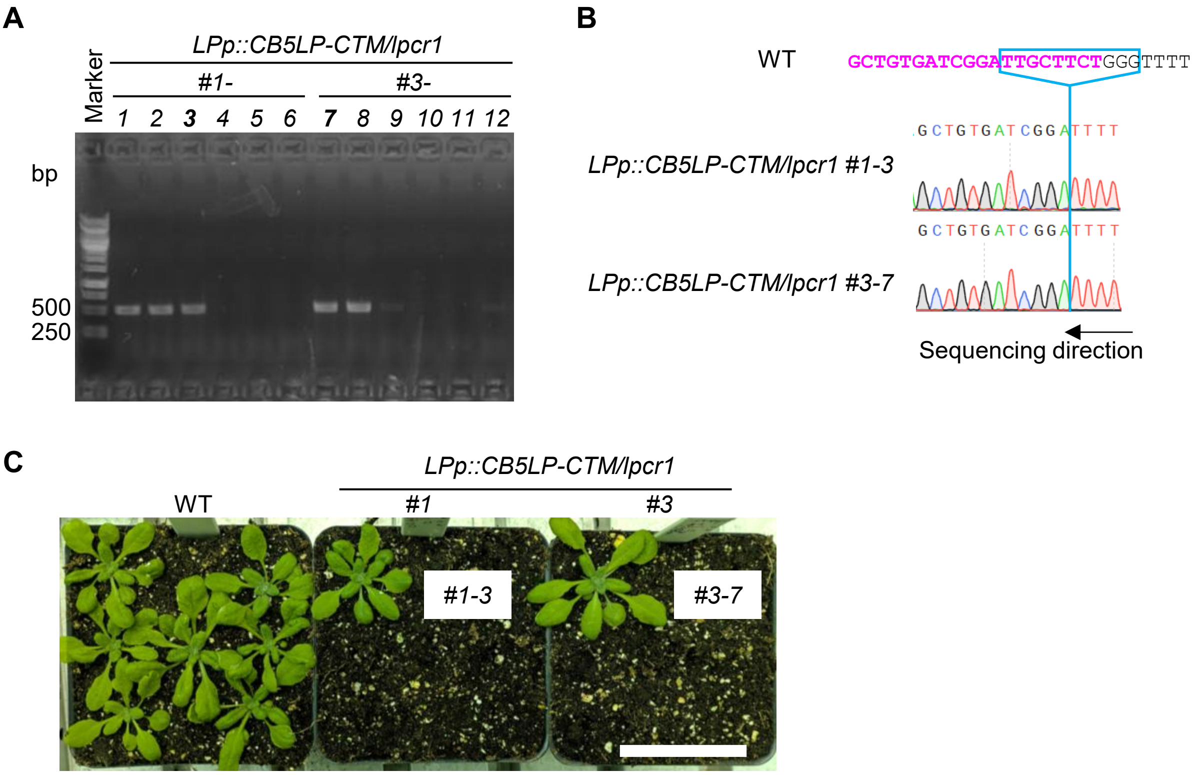

Fig. S16. Confirmation of the *LPp::CB5LP-CTM/lpcr1* transgenic lines.
(**A** and **B**) Genetic background confirmation by PCR genotyping (A) and Sanger sequencing (B). In the T2 generation, six individuals from each of two representative lines (*#1* and *#3*), which survived on 1/2 MS medium containing hygromycin, were selected for PCR genotyping to confirm the presence of the *lpcr1* allele (A). PCR-positive individuals were subsequently subjected to Sanger sequencing to determine their zygosity (homozygous or heterozygous). One plant from each of the two representative lines (*#1* and *#3*) was confirmed to be homozygous for *lpcr1* allele (B). (**C**) Morphology of 3-week-old plants of the two *LPp::CB5LP-CTM/ lpcr1* transgenic lines (*#1-3* and *#3-7*) in a homozygous *lpcr1* background. Scale bar, 5 cm.

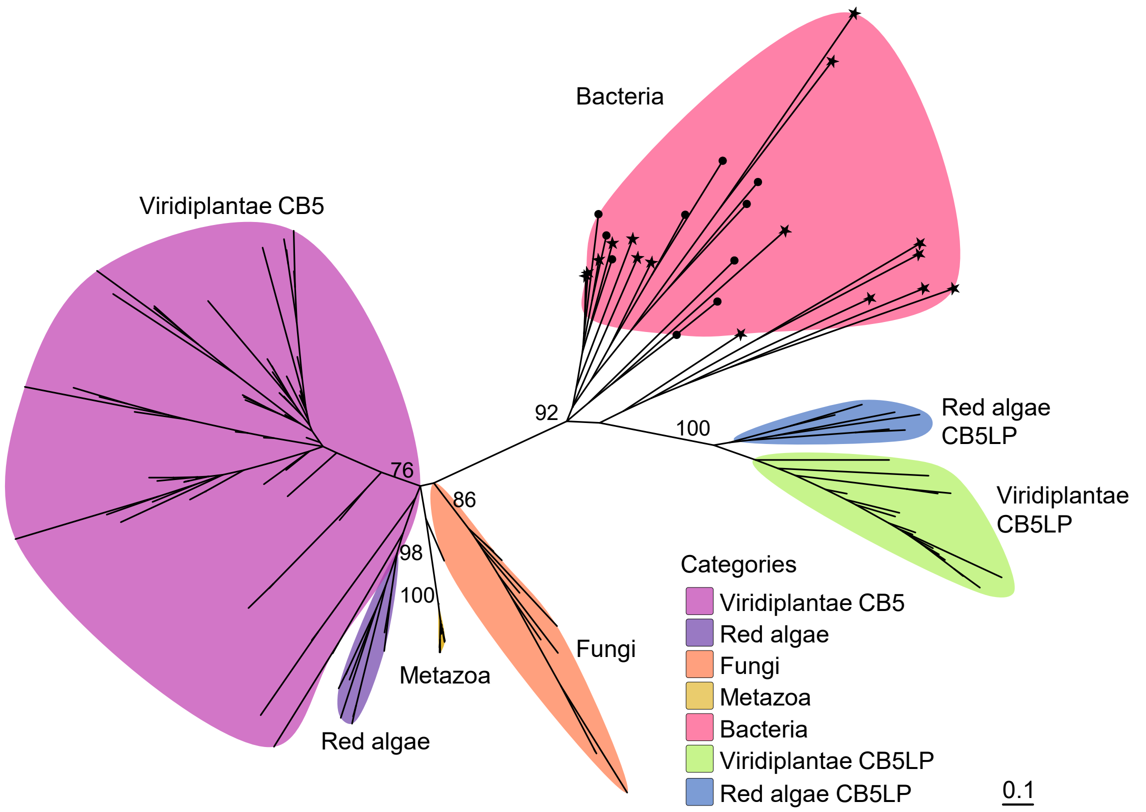

Fig. S17. Phylogeny of CB5 and CB5LP homologs constructed with their cyt-b_5 domain sequences.

CB5 and CB5LP homologs of Viridiplantae and Red algae were obtained using AtCB5D and AtCB5LP as the queries via BLASTP against Phytozome (*Chlamydomonas reinhardtii, Volvox carteri, Marchantia polymorpha, Physcomitrium patens, Selaginella moellendorffii, Ceratopteris richardii, Amborella trichopoda, Oryza sativa,* and *Populus trichocarpa*), FernBase (*Salvinia cucullata*), Genbank (*Porphyridium purpureum, Gracilaria domingensis, Rhodosorus marinus, Cyanidium caldarium, Gracilariopsis chorda* and *Cyanidioschyzon merolae*), ONEKP (*Chara vulgaris, Pinus radiata* and *Ginkgo biloba*) and Solanaceae Genomics Network (*Petunia axillaris*). Metazoa, Fungi and Bacteria CB5 and CB5LP homologs were obtained using AtCB5LP as the query via BLASTP against Clustered nr (nr_clustered) database. Maximum likelihood tree was constructed with the cyt-b_5 domain sequences using IQ-tree2 algorithm (Q.yeast+G4 model selected by ModelFinder, 1000 SH-aLRT tests, and 1000 ultrafast bootstraps). Only major bifurcate bootstrap support values are marked on the main branches. Bacterial proteins with or without a single transmembrane domain are symbolized with stars and dots, respectively. The scale bar indicates average number of amino acid substitutions per site.

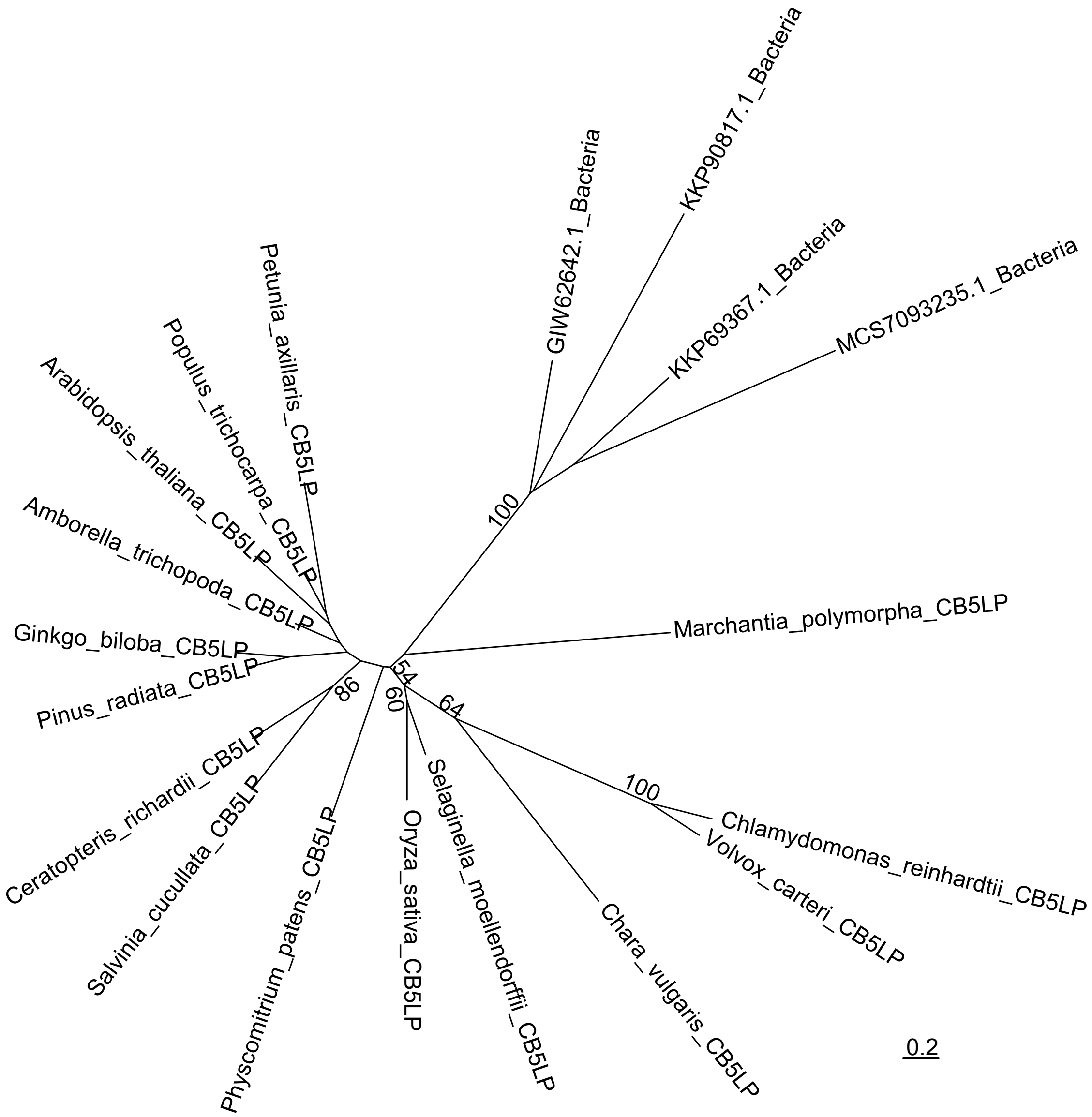

Fig. S18. Phylogeny of Viridiplantae CB5LP homologs.
The Viridiplantae CB5LPs are extracted from Fig. 9. Four bacteria CB5s were included as an out group. Maximum likelihood tree was constructed with the full-length proteins using IQ-tree2 algorithm (Q.yeast+G4 model selected by ModelFinder, 1000 SH-aLRT tests, and 1000 ultrafast bootstraps). Only major bifurcate bootstrap support values are marked on the main branches. The scale bar indicates average number of amino acid substitutions per site.

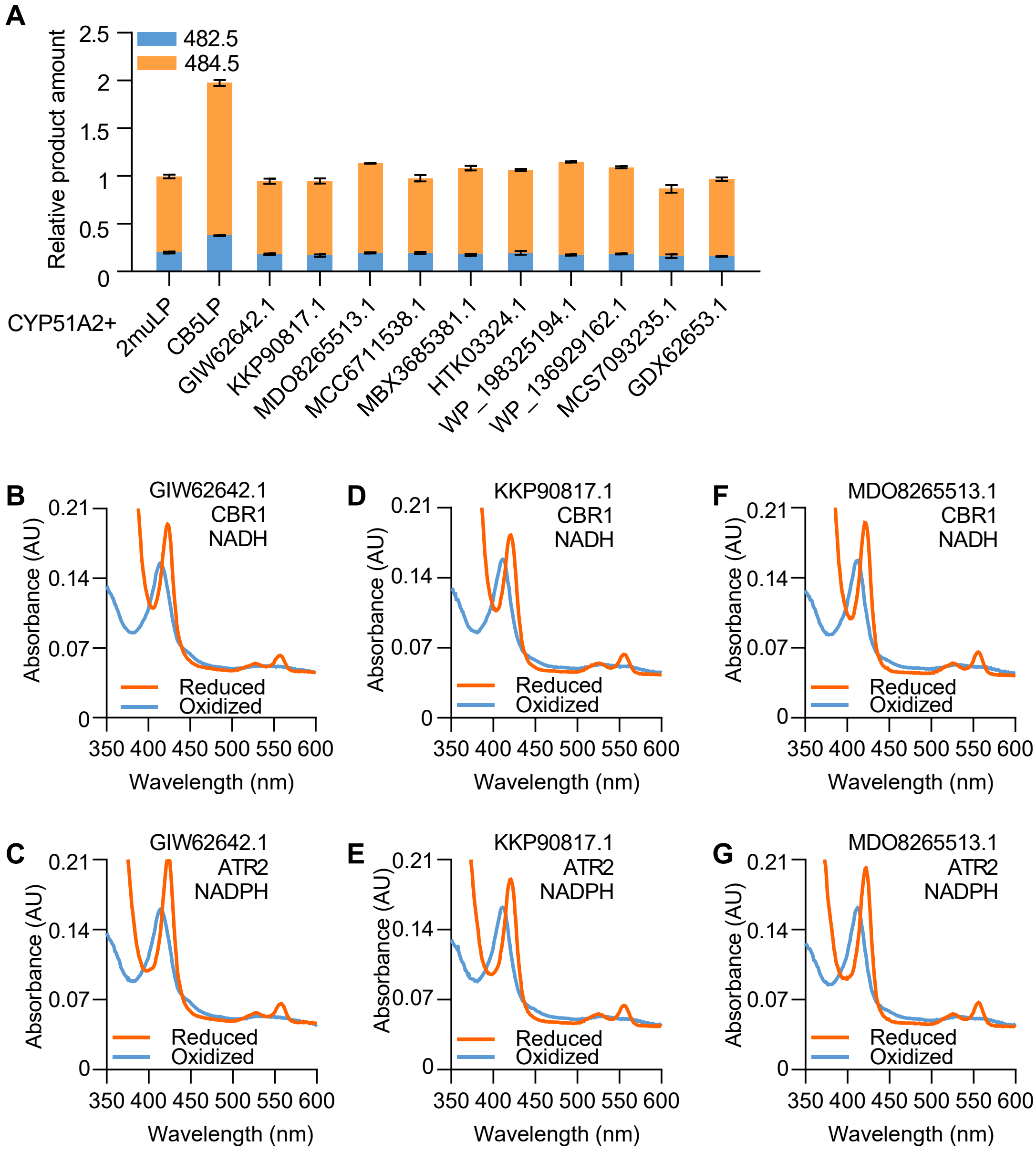

Fig. S19. Characterization of redox activity of bacteria CB5 homologs.

(**A**) Relative activity of CYP51A2 measured using microsomal proteins prepared from yeast cells co-expressing CYP51A2 and the indicated CB5s. NADPH was added as the reductant. Extracted ion chromatogram abundance of *m/z* 482.5 and *m/z* 484.5 were calculated to indicate the activity of CYP51A2. The product amount in reaction of CYP51A2 with 2muLP was set as 1. Data are presented as means ± SD of three technical replicates. (**B** to **G**) UV-visible absorptive spectra of three recombinant bacteria CB5s, GIW62642.1 (B and C), KKP90817.1 (D and E) and MDO8265513.1 (F and G) in the oxidized (blue) or reduced (orange) form. The recombinant CB5 (5 μM) was incubated with CBR1 (86 nM) or ATR2 (282 nM) at room temperature, with the reduction reaction initiated by addition of 50 μM NADH or 50 μM NADPH.

Table S1. Primers used in this study.

Primer's name	Pirmer sequences(5'-3')
**qRT-PCR**	
CB5LP-F	ACAGCAAGAGTGAAGTCGCA
CB5LP-R	GGTCCGAAGAAGCCATCAGT
PP2A-F	TATCGGATGACGATTCTTCGTGCAG
PP2A-R	GCTTGGTCGACTATCGGAATGAGAG
**RT-PCR**	
CB5LP-F/attB1	ACAAGTTTGTACAAAAAAGCAGGCT
CB5LP-R	ATGAAGTTCCCCGATGTAGAAATC
Actin2-F	AACTCTCCCGCTATGTATGTCG
Actin2-R	GAACAAGACTTCTGGGCATCTG
**Genotyping**	
cb5lp-1/pst-lp LP	TGGTGGTTTTGTTTTGTAATGG
cb5lp-1/pst-lp RP	GAGAAAGGAGGAAACTTTGGC
LBb1.3 (LB for SALK line)	ATTTTGCCGATTTCGGAAC
Ds5-2a (LB for pst-lp line)	TCCGTTCCGTTTTCGTTTTTTAC
lpcr1-F (526bp)	GATAGCTGTGATCGGATTTT
lpcr1-R (526bp)	GAGAAAGGAGGAAACTTTGGC
CB5D-F (932bp)	ATGGGCGGAGACGGAAAAG
CB5D-F (932bp)	TCAAGAAGAAGGAGCCTTGG
Cas9-F (528bp)	ggcatccacggggtgccagc
Cas9-R (528bp)	caggaaatggcccctgaac
**Plasmid constructs**	
**pHEE401E-Cas9-CB5LP**	
DT1-CB5LP-BsF(1)	ATATATGGTCTCGATTGCTGTGATCGGATTGCTTCTGTT
DT1-CB5LP-F0(1)	TGCTGTGATCGGATTGCTTCTGTTTTAGAGCTAGAAATAGC
DT2-CB5LP-R0(1)	AAC GCATGATCAAGAATAGCGT CAATCTCTTAGTCGACTCTAC
DT2-CB5LP-BsR(1)	ATTATTGGTCTCGAAAC GCATGATCAAGAATAGCGT C
**pDONR207-CB5LP**	
attB1-CB5LP	GGGG ACA AGT TTG TAC AAA AAA GCA GGC TGC ATGATAGCTGTGATCGGATTGC
attB2-CB5LP	GGG GAC CAC TTT GTA CAA GAA AGC TGG GTC ATGAAGTTCCCCGATGTAGAAATC
**pDONR207-2muCB5LP**	
CB5LPmu1-1	AGAATAGCGTCACCACCAGGAGCTTCTTCAACATAAGAAGTAA
CB5LPmu1-2	TTACTTCTTATGTTGAAGAA GCT CCTGGTGGTGACGCTATTCT
CB5LPmu2-1	ATGTCGAAAACACGAGTGGCAGCTTGAGGTCCGAAGAAGCCAT
CB5LPmu2-2	ATGGCTTCTTCGGACCTCAA GCT GCCACTCGTGTTTTCGACAT
**pDONR207-LPp**	
attB1-CB5LPpro	GGGG ACA AGT TTG TAC AAA AAA GCA GGC TGC aaacttcagaaagtcgaaaaaagccc
attB2-CB5LPpro	GGG GAC CAC TTT GTA CAA GAA AGC TGG GTC ctttctctccttggaaatagcaaaacc
**pDONR207-CB5LP-CTM**	
attB1-CB5LPswap2	GGGG ACA AGT TTG TAC AAA AAA GCA GGC TGC ATGAGATCTAGCAGTGAGCCTG
CB5LPswap2-1	CCGGCACAGTAGCTGTGTCATGAAGTTCCCCGATGTAG
CB5LPswap2-2	CTACATCGGGGAACTTCATGACACAGCTACTGTGCCGG
attB2-CB5LPswap2	GGGGACCACTTTGTACAAGAAAGCTGGGTCTCAAGAAGAAGGAGCCTTGGT
**pDONR207-CYP51A2**	
attB1-CYP51A2	GGGGACAAGTTTGTACAAAAAAGCAGGCTGC ATGGAATTGGATTCGGAGAAC
attB2-CYP51A2	GGGGACCACTTTGTACAAGAAAGCTGGGTC AGAAAGCTGGCGCCTCTTG
**pCR8-tCNX1**	
PCR8-tCNX1-F	ATGATCACGGAACTGATTGAGAAAG
PCR8-tCNX1-R	ATTATCACGTCTCGGTTGCC

**pMDC32-UBIp**	
pMDC32-UBIp-F1036	GTAAAACGACGGCCAGTGCCAAGCTT cgagacttgttcaccaacttg
pMDC32-UBIp-R	CGCGCCTCGAGGGGGGGCCCGGTACC ctgttaatcagaaaaactcagattaatcg
**pMDC32-LPp**	
pMDC32-CB5LPpro-F	GTAAAACGACGGCCAGTGCCAAGCTT aaacttcagaaagtcgaaaaaagccc
pMDC32-CB5LPpro-R	CGCGCCTCGAGGGGGGGCCCGGTACC ctttctctccttggaaatagcaaaacc
**pMDC32-UBIp::CB5LP-mGFP5**	
CB5LP-F	GAGTTTTTCTGATTAACAGGGTACC ATGATAGCTGTGATCGGATTG
CB5LP-R	GTCAGATCTACCATCTCGAGATGAAGTTCCCCGATGTAG
mGFP5-F	CTCGAGATGGTAGATCTGAC
mGFP5-R	GGCGGCCGCTCTAGAACTAG CTCTAGACTCACCTAGGTCAC
**pMDC32-UBIp::CB5LP-mGFP5-TurboID**	
CB5LP-mGFP5-F	GAGTTTTTCTGATTAACAGGGTACC ATGATAGCTGTGATCGGATTG
CB5LP-mGFP5-R	ggggatgggcttgcccat GTGGTGGCTAGCTTTGTATAG
TurboID-F	CTATACAAAGCTAGCCACCAC atgggcaagcccatcccc
TurboID-R	GGCGGCCGCTCTAGAACTAG CTA ctgcagcttttcggcagacc
**pMDC32-UBIp::TurboID-GW**	
TurboID-F	GAGTTTTTCTGATTAACAGGGTACC atgggcaagcccatccc
TurboID-R	CCTCGAGGGGGGGCCCGGTACC tgcttccaccgcctccctgcagcttttcggcagac
**pET28a-sCB5LP/s2muCB5LP**	
pET28a-CB5LP-F(EcoRI)	AGCAAATGGGTCGCGGATCCGAATTC ATGTCTAGCAGTGAGCCTGTAG
pET28a-CB5LP-R(SalI)	TCGAGTGCGGCCGCAAGCTTGTCGAC CTAATGAAGTTCCCCGATG
**pET28a-sGIW62642.1**	
pET28a-sGIW62642.1-F	AGCAAATGGGTCGCGGATCCGAATTC ATGtcgaggcaaaagactacg
pET28a-sGIW62642.1-R	CAGTGGTGGTGGTGGTGGTGCTCGAG tcacttctgaagatctcc
**pET28a-sKKP90817.1**	
pET28a-sKKP90817.1-F	AGCAAATGGGTCGCGGATCCGAATTC ATGaagaaagcagaagctccttc
pET28a-sKKP90817.1-R	CAGTGGTGGTGGTGGTGGTGCTCGAG tcattccttcatgtttctg
**pET28a-sMDO8265513.1**	
pET28a-sMDO8265513.1-F	AGCAAATGGGTCGCGGATCCGAATTC ATGtcgaaagacaagccagag
pET28a-sMDO8265513.1-R	CAGTGGTGGTGGTGGTGGTGCTCGAG tcaattcactaattcaccaatc
**pYeDP60-CYP51A2**	
pYeDP60-CYP51A2-F	AATACACACACTAAATTACCGGATCC ATGGAATTGGATTCGGAGAACA
pYeDP60-CYP51A2-R	AGACATGGGAGATCCCCCGCGAATTC AGAAAGCTGGCGCCTCTTG
**pYeDP60-CYP51A2-T2A-CB5LP/2muLP**	
pYeDP60-CYP51A2-F	AATACACACACTAAATTACCGGATCC ATGGAATTGGATTCGGAGAACA
51A2-T2A-1	gaagacttcctctgccctc AGAAAGCTGGCGCCTCTTG
T2A-F	gagggcagaggaagtcttc
pYeDP60-CB5LP-R	AGACATGGGAGATCCCCCGCGAATTC CTAATGAAGTTCCCCGATGTAG
**pYeDP60-CYP51A2-T2A-CB5LP-CTM**	
pYeDP60-CYP51A2-F	AATACACACACTAAATTACCGGATCC ATGGAATTGGATTCGGAGAACA
T2A-R	AGGGCCGGGATTCTCCTCCA
CB5LP-CTM-F	tggaggagaatcccggccct ATGAGATCTAGCAGTGAGCCTG
CB5LP-CTM-R	AGACATGGGAGATCCCCCGCGAATTC TCAAGAAGAAGGAGCCTTGG
**TOPO-UBIp::CB5LP-PafA**	
CB5LP-PafA-F	gtttttctgattaacactagGTCGAC ATGATAGCTGTGATCGGATTG
CB5LP-PafA-R	caccagatcctcctccgccACTAGTATGAAGTTCCCCGATGTAG

Data S1. The sequence of cloned *CB5LP* gene promoter.

Data S2. List of CB5LP-proximal proteins identified by TurboID-MS.

Data S3. GO pathway enrichment with 1676 enriched proteins (FDR>0.05) using ShinyGo.

Data S4. GO pathway enrichment with 36 enriched cytochrome P450 (FDR>0.05) using ShinyGo.

Data S5. CB5 and CB5LP genes in representative Viridiplantae species and red algae.

Data S6. The top 1000 fungi hits in the BLASTP search with AtCB5LP as query.

Data S7. The top 1000 metazoa hits in the BLASTP search with AtCB5LP as query.

Data S8. The top 1000 bacterial hits in the BLASTP search with AtCB5LP as query.

Data S9. The top 100 hits in the BLAST search with WP_318072142.1 as query.

Data S10. The top 1000 bacterial hits in the BLASTP search with AtCB5D as query.

Data S11. TMHMM analysis of 156 bacterial hits predicted lacking transmembrane domain.

Data S12. The sequences of the 10 bacterial CB5LP genes chosen for co-expression with CYP51A2.

Data S13. Multiple sequence alignments and Newick format trees used in phylogenetic analysis.
